# Fraction of inspired oxygen during general anesthesia for non‐cardiac surgery: Systematic review and meta‐analysis

**DOI:** 10.1111/aas.14102

**Published:** 2022-06-23

**Authors:** Maria Høybye, Peter C. Lind, Mathias J. Holmberg, Maria Bolther, Marie K. Jessen, Mikael F. Vallentin, Frederik B. Hansen, Johanne M. Holst, Andreas Magnussen, Niklas S. Hansen, Cecilie M. Johannsen, Johannes Enevoldsen, Thomas H. Jensen, Lara L. Roessler, Maibritt P. Klitholm, Mark A. Eggertsen, Philip Caap, Caroline Boye, Karol M. Dabrowski, Lasse Vormfenne, Jeppe Henriksen, Mathias Karlsson, Ida R. Balleby, Marie S. Rasmussen, Kim Pælestik, Asger Granfeldt, Lars W. Andersen

**Affiliations:** ^1^ Research Center for Emergency Medicine Aarhus University Hospital Aarhus Denmark; ^2^ Department of Clinical Medicine Aarhus University Aarhus Denmark; ^3^ Department of Surgical Gastroenterology Aalborg University Hospital Aalborg Denmark; ^4^ Department of Cardiology Viborg Regional Hospital Viborg Denmark; ^5^ Department of Anesthesiology and Intensive Care Aarhus University Hospital Aarhus Denmark; ^6^ Prehospital Emergency Medical Services Aarhus Denmark; ^7^ Department of Internal Medicine University Hospital of North Norway Narvik Norway; ^8^ Department of Emergency Medicine, Department of Clinical Research University of Southern Denmark Odense Denmark; ^9^ Department of Anesthesiology and Intensive Care Aalborg University Hospital Aalborg Denmark; ^10^ National Hospital of the Faroe Islands Torshavn Faroe Islands; ^11^ Department of Anesthesiology and Intensive Care Viborg Regional Hospital Viborg Denmark

**Keywords:** complications, fraction of inspired oxygen, general anesthesia, meta‐analysis, outcomes, systematic review

## Abstract

**Background:**

Controversy exists regarding the effects of a high versus a low intraoperative fraction of inspired oxygen (FiO_2_) in adults undergoing general anesthesia. This systematic review and meta‐analysis investigated the effect of a high versus a low FiO_2_ on postoperative outcomes.

**Methods:**

PubMed and Embase were searched on March 22, 2022 for randomized clinical trials investigating the effect of different FiO_2_ levels in adults undergoing general anesthesia for non‐cardiac surgery. Two investigators independently reviewed studies for relevance, extracted data, and assessed risk of bias. Meta‐analyses were performed for relevant outcomes, and potential effect measure modification was assessed in subgroup analyses and meta‐regression. The evidence certainty was evaluated using GRADE.

**Results:**

This review included 25 original trials investigating the effect of a high (mostly 80%) versus a low (mostly 30%) FiO_2_. Risk of bias was intermediate for all trials. A high FiO_2_ did not result in a significant reduction in surgical site infections (OR: 0.91, 95% CI 0.81–1.02 [*p* = .10]). No effect was found for all other included outcomes, including mortality (OR = 1.27, 95% CI: 0.90–1.79 [*p* = .18]) and hospital length of stay (mean difference = 0.03 days, 95% CI −0.25 to 0.30 [*p* = .84). Results from subgroup analyses and meta‐regression did not identify any clear effect modifiers across outcomes. The certainty of evidence (GRADE) was rated as low for most outcomes.

**Conclusions:**

In adults undergoing general anesthesia for non‐cardiac surgery, a high FiO_2_ did not improve outcomes including surgical site infections, length of stay, or mortality. However, the certainty of the evidence was assessed as low.

**Editorial Comment**

Oxygen levels and oxygenation targets in hospitalized patients have received a lot of attention in recent years. In this systematic review, the desirable and undesirable effects of the inspired fraction of oxygen in patients undergoing general anesthesia for non‐cardiac surgery were assessed. Based on low certainty evidence from 25 RCTs, a high fraction of inspired oxygen did not seem to improve outcome, which is in accordance with other published systematic reviews.

## 1 INTRODUCTION

Each year, millions of patients undergo general anesthesia for therapeutic and diagnostic procedures.^1^ During such procedures, anesthetic staff may optimize several ventilator settings with the purpose of reducing post‐operative morbidity and mortality. One key setting is the fraction of inspired oxygen (FiO_2_).

Previous studies have investigated the effects of different levels of FiO_2_ in the acute and intensive care population, however, the effect of different levels of FiO_2_ on post‐operative clinical outcomes in patients undergoing general anesthesia remains unclear. Currently, the World Health Organization recommends the use of an intraoperative FiO_2_ of 80% with the aim of reducing the risk of surgical site infections.^2^ However, the review that constitutes the basis of this recommendation has been criticized for not thoroughly considering the potential adverse effects of a high intraoperative FiO_2_.^3^, ^4^ A high FiO_2_ is not recommended in many other settings such as the emergency department or intensive care unit.^5^, ^6^ Thus, some uncertainty remains regarding this balance of beneficial versus deleterious effects, and it might depend on certain patient and surgical characteristics. Although there are recent systematic reviews on this topic,^7^, ^8^ there are new trials to consider,^9^, ^10^ and these previous reviews have not extensively explored the potential effect heterogeneity between trials.

The goal of this systematic review was to (1) perform a comprehensive review of randomized trials assessing the effect of different levels of intraoperative FiO_2_ during general anesthesia for non‐cardiac surgery on patient‐centered outcomes and (2) explore whether heterogeneity exists according to trial and patient characteristics.

## 2 METHODS

### 2.1 Protocol and registration

This review is part of a series of reviews of clinical trials assessing various respiratory and hemodynamic targets or strategies for patients undergoing general anesthesia for non‐cardiac surgery. This part of the review focuses on FiO_2_ settings or oxygenation targets (PaO_2_ or oxygen saturation), and findings for other targets (i.e., goal‐directed hemodynamic therapy,^11^ blood pressure, and ventilation) are reported separately. As no trials on oxygenation targets were identified, we focus on FiO_2_ in the remainder of the manuscript. The protocol was uploaded to figshare.com on June 11, 2020, and updated on August 19, 2020 and is provided in the Appendix S1. The reporting of this review followed the Preferred Reporting Items for Systematic Reviews and Meta‐Analyses (PRISMA) guidelines.^12^ The PRISMA checklist is provided in the Appendix S1.

### 2.2 Eligibility criteria and outcomes

The research question was framed according to the PICO format: (1) In adults undergoing general anesthesia with invasive mechanical ventilation for non‐cardiac surgery (P), does a specific intraoperative FiO_2_ (I), as compared to a different intraoperative FiO_2_ (C) result in better clinical outcomes (O); (2) Trials including very short duration of anesthesia (e.g., for electroconvulsive therapy), cesarean sections, interventional radiology, and surgery requiring one‐lung ventilation were excluded.

Only randomized clinical trials were included, including quasi‐randomized (e.g., intervention assignment by day or week) trials as well as cluster‐randomized trials. Randomized cross‐over trials, where the cross‐over occurred within individual patients, were not included. Only trials published as full‐text articles and in English language were included. There was no limitation regarding the year of publication.

The main clinically relevant outcomes reported were mortality, hospital length of stay, and surgical site infection. Additional reported outcomes included other post‐operative complications (e.g., pulmonary complications and cardiac complications), as well as other patient‐centered outcomes (e.g., intensive care unit admission and quality of life). Additional details about the categories and definitions used for post‐operative complications are provided in the Supplement. Trials focusing only on physiological or surrogate outcomes were not included. Outcomes related to post‐operative pain, nausea, and vomiting will be reported in a separate manuscript.

### 2.3 Information sources and search strategy

We searched PubMed and Embase on May 28, 2020, July 24, 2020, and March 8, 2021. The search was updated on March 22, 2022. The search strategy reflects that the current review on FiO_2_ is part of a series of reviews, with a combined search strategy evaluating multiple respiratory and hemodynamic targets during general anesthesia. The search included a combination of various text and indexing search terms for general anesthesia or surgery and the various targets. To identify randomized trials, the Cochrane sensitivity‐maximizing search strategy was used.^13^ The full search strategy for both databases is provided in the protocol. The updated search strategy is provided in the Supplement.

To identify registered ongoing trials, the International Clinical Trials Registry Platform was searched on April 5, 2021 and again on June 28, 2021. Additional details are provided in the Supplement.

### 2.4 Study selection

Pairs of two reviewers independently screened all titles and abstracts retrieved from the systematic searches. Any disagreements regarding inclusion or exclusion were resolved via discussion between the reviewers and with a third reviewer as needed. Two reviewers then independently reviewed the full texts of all potentially relevant publications passing the first level of screening. Any disagreement regarding eligibility was resolved via discussion. The Kappa values for inter‐observer variance were calculated. In case of poor inter‐reviewer agreement (i.e., κ < 0.4), a third reviewer reviewed all excluded titles and abstracts to ensure optimized sensitivity. The bibliographies of included articles as well as recent reviews^14^ were reviewed for potential additional relevant manuscripts.

### 2.5 Data collection

Two reviewers, using a pre‐defined standardized data extraction form, extracted data from individual manuscripts. Any discrepancies in the extracted data were resolved via discussion.

### 2.6 Risk of bias in individual studies

Two investigators independently assessed the risk of bias for the included trials using the revised Cochrane risk‐of‐bias tool for randomized trials.^15^ Disagreements were resolved via discussion. Risk of bias was assessed for each outcome within a trial but is reported at the trial level as the highest risk of bias score across all outcomes. If the bias varied according to the outcomes, this was noted.

### 2.7 Data synthesis and confidence in cumulative evidence

Included trials were assessed for clinical (i.e., participants, interventions, comparators), methodological (ie study design or risk of bias), and statistical heterogeneity. If no major clinical or methodological heterogeneity was identified, meta‐analyses were performed using Review Manager 5.4.1 (Cochrane Collaboration, Nordic Cochrane Centre). For dichotomous variables, Peto's odds ratio (OR) method was used for all meta‐analyses, including meta‐regression. This method was used as many of the outcomes were infrequent or occurred in zero patients in one of the treatment arms.^16^, ^17^ Results for the dichotomous variables are reported as ORs with 95% confidence intervals. For continuous variables (ie hospital length of stay), the inverse variance method with random effects was used for meta‐analyses. Results from these analyses are reported as the mean difference with 95% confidence intervals. Several manuscripts reported hospital length of stay using medians and quartiles. In order to use these results in meta‐analyses, we estimated means and standard deviations, assuming normality of the data.^18^

Based on the available data, we conducted several post hoc subgroup analyses according to surgical characteristics. These included ≥50% versus <50% of the included patients requiring acute surgery and ≥50% versus <50% of the included patients undergoing abdominal surgery. Subgroup analyses according to other patient‐ and interventional characteristics, for example laparoscopic versus non‐laparoscopic surgery, were not feasible in this context due to insufficient trials reporting relevant data.

Meta‐regression was performed to evaluate the relationship between selected potential continuous moderators and the outcomes of mortality, hospital length of stay, and surgical site infection. Only comparisons with at least 10 trials were considered. Moderators included median year of patient inclusion, duration of surgery (in minutes), and sample size, as well as mortality and hospital length of stay (in days) in the control group as a reflection of the illness severity in the underlying trial population. The latter two analyses should be interpreted with caution due to the potential for regression to the mean.^19^, ^20^ Results are presented using bubble plots with the size of each bubble corresponding to the inverse variance of the effect size in each trial. Meta‐regression was performed using STATA version 16 (StataCorp LP).

We performed sensitivity analyses excluding trials with a FiO_2_ level different from 80% and/or 30%. To assess for potential publication bias for the primary outcomes, funnel plots were created and visually interpreted.

### 2.8 Cumulative evidence (GRADE)

The certainty of the overall evidence for a given comparison and outcome was assessed using the Grading of Recommendations Assessment, Development and Evaluation (GRADE) methodology and classified within one of four categories: very low, low, moderate, or high certainty of evidence.^21^ Additional details are provided in the Supplement. GRADEpro (McMaster University, 2020) was used for drafting of the GRADE table.

## 3 RESULTS

### 3.1 Overview

The systematic search yielded 23,936 unique titles/abstracts, of which 23,393 were excluded during the initial screening (κ = 0.61, Figure S1). Five hundred and forty‐three full manuscripts were screened, of which 34 manuscripts investigated different levels of intraoperative FiO_2_ with no trials investigating other oxygen related targets (eg oxygen saturation). Four of these trials^22^, ^23^, ^24^, ^25^ were excluded due to data irregularities.^26^ Three additional manuscripts were identified by reviewing bibliographies. A total of 33 manuscripts published between 2000 and 2022 were therefore included. The manuscripts represented 24 separate randomized trials,^22^, ^23^, ^24^, ^25^, ^27^, ^28^, ^29^, ^30^, ^31^, ^32^, ^33^, ^34^, ^35^, ^36^, ^37^, ^38^, ^39^, ^40^, ^41^, ^42^, ^43^, ^44^, ^45^, ^46^, ^47^ one alternating intervention trial,^48^ and eight post hoc or subgroup analyses of these trials.^49^, ^50^, ^51^, ^52^, ^53^, ^54^, ^55^, ^56^ Data on a total of 15,032 patients were included. We additionally identified 25 ongoing or unpublished trials with details provided in the (Appendix S1).

All but six trials compared an intraoperative FiO_2_ of 80% to an intraoperative FiO_2_ of 30%. Two trials investigated an intraoperative FiO_2_ of 100%^45^ or 50%^32^ in the intervention arm, one trial an FiO_2_ of 80% in the intervention arm and 40% in the control arm,^9^ one trial an FiO_2_ of 65% in the intervention arm and 35% in the control arm,^57^ and finally two trials an FiO_2_ of 33%^37^ or 35%^29^ FiO_2_ in the control arm. Most of the trials were small (median sample size = 252) with only 32% (*n* = 8) including 500 patients or more. A total of 64% of the trials (*n* = 16) primarily included patients undergoing abdominal surgery.

An overview of the included manuscripts is provided in Table 1, and patient and surgical characteristics are provided in the Table S2.

**TABLE 1:** Characteristics of included manuscripts

Trial	Country	Years of patient inclusion	Main inclusion criteria	No. of patients	Intervention FiO_2_	Control FiO_2_
Kotani (2000)^45^	Japan	NR	Scheduled to undergo orthopedic surgery >6 h	60	100%	30%
Greif (2000)^36^	Austria, Germany	1996–1998	Age 18–80, elective open colorectal resection	500	80%	30%
Purhonen (2002)^71^	Finland	NR	Female, ASA I‐II, ambulatory gynecologic laparoscopy	100	80%	30%
Pryor (2004)^29^	USA	2001–2003	Major open abdominal surgery	165	80%	35%
Mayzler (2005)^38^	Israel	2001–2002	Elective colorectal surgery for malignant disease	38	80%	30%
Belda (2005)^32^	Spain	2003–2004	Elective colorectal resection	300	50%	30%
Myles (2007)^40^	Multiple	2003–2004	Expected duration >2 h, anticipated length of hospital stay ≥3 days	2050	80%	30%^a^
Meyhoff (2009)^39^	Denmark	2006–2008	Abdominal laparotomy	1400	80%	30%
McKeen (2009)^28^	Canada	2003–2005	Ambulatory laparoscopic tubal ligation, ASA I‐II	304	80%	30%
Bickel (2011)^33^	Israel	2006–2009	ASA I‐IV, open appendectomy for acute appendicitis	210	80%	30%
Thibon (2012)^43^	France	2003–2007	Elective abdominal, gynecological, or breast surgery	434	80%	30%
Staehr (2012)^56^	Denmark	2008	Scheduled for laparotomy for ovarian cancer	35	80%	30%
Meyhoff^b^ (2012)^49^	Denmark	2006–2008	Abdominal laparotomy	1400	80%	30%
Stall (2013)^42^	USA	2007–2010	High‐energy lower extremity fracture	228	80%	30%
Chen (2013)^46^	China	2009–2011	Elective open colorectal surgery	60	80%	30%
Meyhoff^b^ (2014)^50^	Denmark	2006–2008	Abdominal laparotomy	1400	80%	30%
Kurz (2015)^27^	Ireland, Switzerland, Austria, USA	2002–2007	Age ≤80, elective colorectal resection expected to last 2–6 h	586	80%	30%
Wasnik (2015)^47^	India	NR	Open appendectomy for acute appendicitis	64	80%	30%
Fonnes (2016)^51^	Denmark	2006–2008	Abdominal laparotomy	1377	80%	30%
Chiang (2017)^34^	New Zealand	2009–2011	Infrainguinal bypass surgery	80	80%	30%
Kurz (2017)^48^	USA	2013–2016	Colorectal surgery	5749	80%	30%
Mayank (2018)^37^	India	NR	Elective colorectal surgery, expected duration >1 h	94	80%	33%
Kongebro^c^ (2018)^52^	Denmark	2006–2008	Abdominal laparotomy	1386	80%	30%
Alvandipour (2018)^31^	Iran	NR	Colorectal surgery	85	80%	30%
Ruetzler^d^ (2019)^53^	USA	2013–2016	Colorectal surgery	4481	80%	30%
Cohen^e^ (2019)^54^	USA	2013–2016	Colorectal surgery	5056	80%	30%
Ferrando (2019)^35^	Spain	2017–2018	BMI < 35, major abdominal surgery, expected duration >2 h	717	80%	30%
Li (2020)^44^	China	2018	Age ≥18, ASA I‐III, elective abdominal surgery expected to last >2 h	252	80%	30%
Jiang^e^ (2021)^55^	USA	2013–2016	Colorectal surgery	3471	80%	30%
Lin (2021)^9^	China	2018–2020	Laparoscopic surgery for gastric and colorectal malignancies, ASA I‐III, NYHA I‐II, age 65–85 years	630	80%	40%
Park (2021)^57^	South Korea	2020	Age ≥50 years, ASA I‐III, elective abdominal surgery, expected duration >1 h	190	60%	35%
Reiterer (2021)^72^	Austria	2017–2019	Elective moderate to high‐risk abdominal surgery, expected duration >2 h, age >45 years, cardiovascular risk	260	80%	30%
Holse (2022)^58^	Denmark	2018–2020	Cardiovascular risk factors, age ≥45 years	576	80%	30%

Abbreviations: FiO_2_, fraction of inspired oxygen; NR, not reported; GA, general anesthesia; PONV, postoperative nausea and vomiting; NYHA, New York Heart Association Functional Classification.

^a^ ± N_2_O.

^b^ Follow‐up study of Meyhoff (2009).^39^

^c^ Post‐hoc analysis of Meyhoff (2009).^39^

^d^ Subanalysis of Kurz (2017).^48^

^e^ Post‐hoc analysis of Kurz, (2017).^48^

**FIGURE 1:**
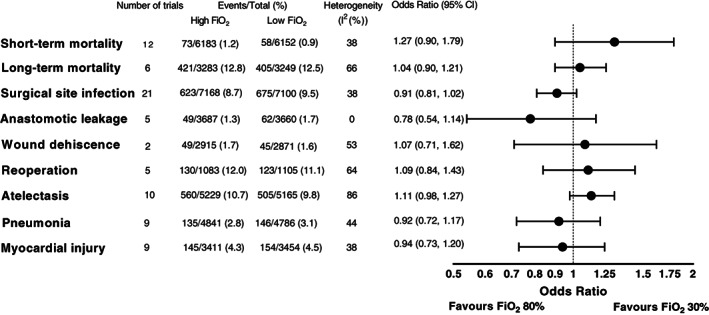
Overview of results from meta‐analyses of binary outcomes. FiO_2_, fraction of inspired oxygen; CI, confidence interval.

The included trials reported various outcomes (Table S3), of which we performed meta‐analyses on a total of nine outcomes (Figure 1). The remaining outcomes were not eligible for meta‐analyses as a limited number of trials reported these outcomes or due to very heterogeneous definitions. The outcomes included in the meta‐analysis were short‐ and long‐term mortality, hospital length of stay, surgical site infection, anastomotic leakage, wound dehiscence, need for reoperation, atelectasis, pneumonia, and myocardial injury/infarction.

All trials were assessed as having an overall intermediate risk of bias (Table S4).

### 3.2 Mortality

We identified 16 trials that addressed the effect of high versus low intraoperative FiO_2_ on mortality (Table S3). Of these, 12 reported short‐term mortality, which included in‐hospital mortality, mortality during the trial period, and 7‐, 15‐, and 30‐day mortality, and six trials reported long‐term mortality with median follow‐up ranging from 180 days to 4 years across trials. A total of 12,335 and 6532 patients were included in the meta‐analysis on short‐ and long‐term mortality, respectively.

**FIGURE 2:**
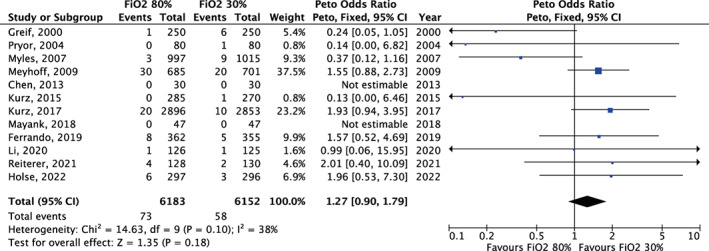
Meta‐analysis for short‐term mortality. FiO_2_, Fraction of inspired oxygen; CI, confidence interval.

For both short‐ and long‐term mortality, we found no difference in survival between high and low FiO_2_ (OR = 1.27, 95% CI 0.90–1.79 [*p* = .18] and OR = 1.04, 95% CI 0.90–1.21 [*p* = .60], respectively (Figure 1 and Figure 2, Figure S2]). The results from the subgroup analyses for long‐term mortality were similar but was limited by the low number of trials (Figure S3). In an analysis of overall mortality including 13,293 patients, there was no difference between high and low FiO_2_ (OR = 1.03, 95% CI 0.75–1.40 [*p* = .87], Figure S4).

Results from meta‐regressions are provided in Figures S5–S7 and Table S5. There was no clear effect measure modification according to short‐term mortality in the control group or sample size. More recent trials, as compared to older trials, favored a lower FiO_2_. Specifically, each subsequent median year of patient inclusion increased the effect size by 1.08 (95% CI 1.02–1.13 [*p* = .005]), indicating a more harmful effect of high oxygen compared to low oxygen.

In the funnel plot, we found no clear evidence of publication bias for short‐term mortality, although the interpretation was limited by the low number of trials (Figure S8).

The results were similar in the sensitivity analysis (Table S6).

### 3.3 Hospital length of stay

Seventeen trials reported data on hospital length of stay for a total of 9064 patients (Table S3). Two trials included patients scheduled for ambulatory surgery where hospital length of stay was only a few hours, and another trial only reported the outcome's variation as a range. These trials were not included in the meta‐analysis. We found no evidence of a difference in hospital length of stay between the high FiO_2_ group and the low FiO_2_ group (mean difference = 0.03 days, 95% CI ‐0.25 to 0.30, [*p* = .84], Figure S9). No differences between groups were found in subgroup analyses (Figures S10 and S11).

In the meta‐regression, there were no clear effect measure modification according to median year of patient inclusion, short‐term mortality in the control group, length of stay in the control group, and duration of surgery (all *p* > .05 [Figures S12–S16 and Table S5]). Larger as compared to smaller trials favored a higher FiO_2_. Specifically, each additional 100 subjects changed the mean difference in the effect size by 0.03 (95% CI, −0.06 to −0.00 [*p* = .03]) in favor of a higher FiO_2_.

In the funnel plot, we found no clear evidence of publication bias, although the interpretation was limited by the low number of trials (Figure S17).

The result was similar in the sensitivity analysis (Table S6).

### 3.4 Surgical site infection

**FIGURE 3:**
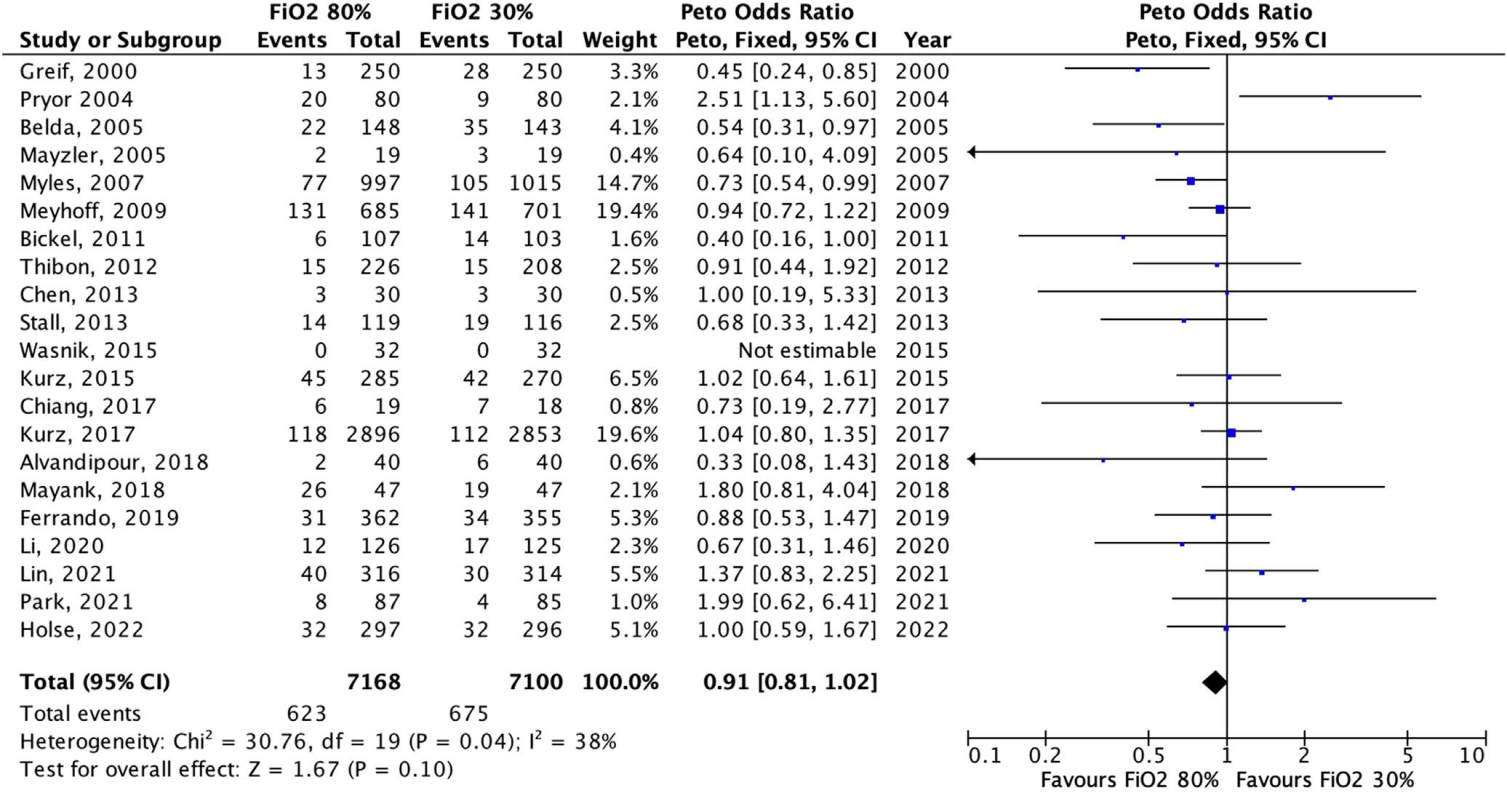
Meta‐analysis for surgical site infection. FiO_2_, Fraction of inspired oxygen; CI, confidence interval.

Twenty‐one trials reported data on postoperative surgical site infection for a total of 14,268 patients (Table S3). In the meta‐analysis, high FiO_2_ versus low FiO_2_ did not result in a significant decrease in the risk of surgical site infection (OR = 0.91, 95% CI 0.81–1.02 [*p* = .10], Figure 3).

In the subgroup on ≥50% versus <50% acute surgery, we found the most prominent effect of high FiO_2_ in the subgroup with ≥50% acute surgery (OR = 0.55, 95% CI 0.31–0.98 [*p* = .04]) versus <50% acute surgery (OR = 0.92, 95% CI 0.82–1.04 [*p* = .20], Figure S18), although the test for subgroup differences was not significant (*p* = .09). There was no subgroup difference according to ≥50% versus <50% abdominal surgery (Figure S19).

In the meta‐regression, there were no clear effect measure modifiers (all *p* > .05 Figures S20–S24 and Table S5]).

In the funnel plot, we found no clear evidence of publication bias (Figure S25).

The result was similar in the sensitivity analysis (Table S6).

### 3.5 Postoperative complications

In addition to surgical site infection, some of the included trials reported data on other postoperative surgical complications, including five on anastomotic leakage for 7347 patients, two on wound dehiscence for 5786 patients, and five on reoperation for 2188 patients. We found no evidence of differences in the incidence of anastomotic leakage (OR = 0.78, 95% CI 0.54–1.14 [*p* = .21], Figure S26), wound dehiscence (OR = 1.07, 95% CI 0.71–1.62 [*p* = .74], Figure S27), or reoperation (OR = 1.09, 95% CI 0.84–1.43 [*p* = .51], Figure S28) between the trial groups. Results were similar in the subgroup analyses for reoperation, although this analysis was limited by the low number of trials (Figure S29).

Ten trials reported data on atelectasis for 10,394 patients and nine trials on pneumonia for 9627 patients. The pooled estimates did not indicate a difference between groups (OR = 1.11, 95% CI 0.98–1.27 [*p* = .11], Figure S30 and OR = 0.92, 95% CI 0.72–1.17 [*p* = .50], Figure S31). The same was evident in the subgroup analyses, although these analyses were limited by the low number of trials and patients Figures S32 and S33).

Nine trials reported data on myocardial injury/infarction for 6865 patients. In the meta‐analysis, we found no difference between groups (OR = 0.94, 95% CI 0.73–1.20, Figure S34 [*p* = .61]). Similar results were found in the subgroup analysis (Figure S35) although this analysis was limited by the low number of trials.

Sensitivity analyses on these postoperative complications generally showed similar results to the primary analyses, although they were limited by the low number of trials (Table S6).

### 3.6 Cumulative evidence (GRADE)

Using GRADE, the overall certainty for most of the included outcomes was assessed as low. For anastomotic leakage and hospital length of stay the overall certainty was assessed as moderate. The GRADE assessment is found in Table S7.

## 4 DISCUSSION

This systematic review included 33 manuscripts describing results from 25 separate trials with almost 15,000 patients. The trials all investigated the effect of high (mostly 80%) versus low (mostly 30%) FiO_2_ on various clinical and postoperative outcomes. In the meta‐analyses, there was no significant difference between a high and a low FiO_2_ for all outcomes including surgical site infection, length of stay, and mortality. The overall certainty in the evidence was considered low for most of the outcomes.

The included trials were generally small with only eight trials including 500 patients or more^9^, ^27^, ^35^, ^36^, ^39^, ^40^, ^48^, ^58^ and only three trials including more than 1000 patients.^39^, ^40^, ^48^ A noticeable proportion of the trials did not report patient and surgical characteristics deemed crucial for determining potential clinical heterogeneity (e.g., ASA score, length of surgery). Moreover, in some cases, the outcomes were poorly and heterogeneously defined, leading to difficulties in including the outcomes in meta‐analyses. However, there was only little between‐study heterogeneity in the interventions and comparators used, as most of the trials compared an FiO_2_ of 80% to 30%.

No trials were assessed as having a low risk of bias. All the included trials had an intermediate risk of bias. This was largely because no trials had intraoperative blinding of the clinical team performing the intervention, which cannot rule out a risk of bias due to the possibility of deviations from the intended interventions.

The trade‐off between the beneficial and potential detrimental effects of hyperoxemia has been subject to vigorous debate and research. The potential beneficial effects of hyperoxemia on surgical site infection are twofold. First, the risk of surgical site infection is inversely related to tissue oxygenation in observational settings.^59^, ^60^ It is therefore plausible that ensuring a high tissue oxygenation will decrease the risk of surgical site infection. Second, it has been shown in the laboratory that the intrinsic ability of the immune system to eliminate pathogens is highly oxygen dependent.^61^ As such, tissue hyperoxygenation might promote phagocytosis and consequently prevent infection. In the current review, there was no significant improvement in surgical site infection with a high FiO_2_. This is in contrast with findings in previous reviews,^14^, ^62^ and likely reflects the inclusion of newer trials. However, the point estimate in the current meta‐analysis suggested fewer surgical site infections with a high FiO_2_ but there was some heterogeneity in the results from the included trials and wide confidence intervals (Figure 2), and the overall certainty in the evidence was therefore rated as low. Although there was some indication that the results suggesting benefit were primarily driven by older trials, we did not find a significant association between the year of patient inclusion and the effect size in meta‐regression (Table S5). Based on this, we did not downgrade the evidence for indirectness. Lastly, the effect size on surgical site infection was relatively small. The importance of this finding, given the uncertainty and especially in the context of the remaining outcomes, is therefore unclear.

On the other hand, a high FiO_2_ might potentially promote atelectasis,^63^, ^64^ which in turn might promote respiratory infection. Furthermore, exposure to a high oxygen concentration might cause cellular damage and lung injury through the formation of reactive oxygen species,^65^ an effect that is evident in animal models after only short exposure to high levels of oxygen.^66^ There is some evidence of deleterious effects of high levels of oxygen in the acute and intensive care population, where it has been associated with worse clinical outcomes in some studies,^67^ although the pooled evidence is inconclusive.^68^ Recent evidence suggests that in patients with acute hypoxemic respiratory failure, there is no effect on mortality with higher versus lower oxygenation targets.^69^ In the present review, as well as in previously published reviews,^14^, ^62^, ^70^ no adverse effects of high levels of oxygen on pulmonary (including atelectasis), cardiovascular, and clinical complications (ie mortality, length of hospital stay) were evident. However, for most of these outcomes, the certainty of evidence was low (Table S7).

While some trials have suggested that a high FiO_2_ could result in increased mortality,^39^, ^48^ we found no difference in short‐ or long‐term mortality in the meta‐analyses. Given the relatively low mortality in the included population, it is difficult to exclude a clinically important difference in mortality between the groups. For example, if a trial was designed to detect a difference in short‐term mortality of 1.0% versus 1.5%, approximately 15,000 to 20,000 patients would have to be included.

This systematic review provides an update on intraoperative FiO_2_ and was performed using rigorous methodology. The review differs from previous reviews by including newly published trials and by considering the effect of the intervention in subgroups that have not previously been investigated. Future trials should focus on reporting and consistently defining clinically relevant and patient‐centered outcomes, and in this context aim at including cohorts of considerable size to allow for detection of meaningful differences in these outcomes. Furthermore, future trials should aim at blinding all personnel involved in assessment of the outcomes as this would minimize the risk of bias.

This review has some limitations. First, specific outcomes and their definitions were not prespecified in the protocol. This was done to capture all the clinically relevant outcomes that were reported in the included trials. However, this approach might have introduced some subjectivity into which outcomes were included in the manuscript. Second, the subgroup analyses performed were not prespecified and were based on the available data provided in the included trials. For many of the subgroups, very few trials were available. Third, there was no limit on trial publication year leading to pooling of trials spanning more than 20 years. We did, however, perform meta‐regression to assess the effect of publication year. Fourth, patient and surgical characteristics as well as outcomes were poorly, or in some cases not, defined leading to some degree of subjectivity and unreliability in classifying trials and in meta‐analysis inclusion. Fifth, while the intervention was similar across trials, there was some heterogeneity in the included patient populations and outcomes. While we explored this heterogeneity in subgroup analyses and meta‐regression, the results from the meta‐analyses should be carefully interpreted. Sixth, we did not contact trial authors for additional information about outcomes that were not reported. Lastly, we did not include unpublished trials, including trials only published as abstracts. As such, we might have missed relevant trials.

In adults undergoing general anesthesia for non‐cardiac surgery, a high FiO_2_ does not improve clinically relevant postoperative outcomes. For most outcomes, the certainty in the evidence was assessed as low and it therefore remains unclear whether applying a high FiO_2_ is beneficial or harmful. Our findings do not support current WHO guidelines to use a FiO_2_ of 80%.^2^

## AUTHOR CONTRIBUTIONS

LWA, AG, and MJH were involved in study conception and design. All authors were involved in data acquisition, data interpretation, and critical revision of the manuscript for important intellectual content. MH, PCL, MH, and LWA were involved in data analysis. MH, PCL, and LWA were involved in drafting the manuscript. All authors reviewed the results and approved the final version of the manuscript.

## CONFLICT OF INTEREST

None of the authors have any conflict of interest.

## Supporting information


**Appendix S1** Supporting Information
