# Supplementary material for: Fraction of inspired oxygen during general anesthesia for non‐cardiac surgery: Systematic review and meta‐analysis
Source: Acta Anaesthesiol Scand. 2022 Jun 23;66(8):923–33. doi: 10.1111/aas.14102 (PMC9543529; doi:10.1111/aas.14102)
Supplement: Supplementary file 1 — Appendix S1 Supporting Information [file AAS-66-923-s001.docx]

**SUPPLEMENT MATERIAL**

**Fraction of Inspired Oxygen During General Anesthesia for Non-Cardiac Surgery: Systematic Review and Meta-Analysis**

CONTENT

[SUPPLEMENTAL METHODS 4](#_Toc101437250)

[Outcomes 4](#_Toc101437251)

[Risk of bias assessment 6](#_Toc101437252)

[Search for ongoing trials 8](#_Toc101437253)

[Updated search for studies 8](#_Toc101437254)

[GRADE evaluation 9](#_Toc101437255)

[TABLES 12](#_Toc101437256)

[Table S1: Registered randomized trials comparing oxygen targets 12](#_Toc101437257)

[Table S2: Patient and surgical characteristics in included manuscripts 15](#_Toc101437258)

[Table S3: Reported outcomes in the included manuscripts^a^ 17](#_Toc101437259)

[Table S4: Bias assessment of included manuscripts 20](#_Toc101437260)

[Table S5. Meta regression analyses 22](#_Toc101437261)

[Table S6: Sensitivity analyses 24](#_Toc101437262)

[Table S7: GRADE assessment 26](#_Toc101437263)

[FIGURES 28](#_Toc101437264)

[Figure S1: PRISMA diagram 28](#_Toc101437265)

[Figure S2: Long-term mortality, meta-analysis 29](#_Toc101437266)

[Figure S3: Long-term mortality, abdominal surgery subgroup analysis 30](#_Toc101437267)

[Figure S4: Overall mortality (closets to 30 days), meta-analysis 31](#_Toc101437268)

[Figure S5: Bubble plot for meta-regression of short-term mortality and median year of patient inclusion 32](#_Toc101437269)

[Figure S6: Bubble plot for meta-regression of short-term mortality and mortality in the control group 33](#_Toc101437270)

[Figure S7: Bubble plot for meta-regression of short-term mortality and sample size 34](#_Toc101437271)

[Figure S8: Funnel plot short-term mortality 35](#_Toc101437272)

[Figure S9: Hospital length of stay, meta-analysis 36](#_Toc101437273)

[Figure S10: Hospital length of stay, acute surgery subgroup analysis 37](#_Toc101437274)

[Figure S11: Hospital length of stay, abdominal surgery subgroup analysis 38](#_Toc101437275)

[Figure S12: Bubble plot for meta-regression of length of stay and median year of patient inclusion 39](#_Toc101437276)

[Figure S13: Bubble plot for meta-regression of length of stay and mortality in the control group 40](#_Toc101437277)

[Figure S14: Bubble plot for meta-regression of length of stay and length of stay in the control group 41](#_Toc101437278)

[Figure S15: Bubble plot for meta-regression of length of stay and duration of surgery 42](#_Toc101437279)

[Figure S16: Bubble plot for meta-regression of length of stay and sample size 43](#_Toc101437280)

[Figure S17: Funnel plot hospital length of stay 44](#_Toc101437281)

[Figure S18: Surgical site infection, acute surgery subgroup analysis 45](#_Toc101437282)

[Figure S19: Surgical site infection, abdominal surgery subgroup analysis 46](#_Toc101437283)

[Figure S20: Bubble plot for meta-regression of surgical site infection and median year of patient inclusion 47](#_Toc101437284)

[Figure S21: Bubble plot for meta-regression of surgical site infection and mortality in the control group 48](#_Toc101437285)

[Figure S22: Bubble plot for meta-regression of surgical site infection and length of stay in the control group 49](#_Toc101437286)

[Figure S23: Bubble plot for meta-regression of surgical site infection and duration of surgery 50](#_Toc101437287)

[Figure S24: Bubble plot for meta-regression of surgical site infection and sample size 51](#_Toc101437288)

[Figure S25: Funnel plot surgical site infection 52](#_Toc101437289)

[Figure S26: Anastomotic leakage, meta-analysis 53](#_Toc101437290)

[Figure S27: Wound dehiscence, meta-analysis 54](#_Toc101437291)

[Figure S28: Reoperation, meta-analysis 55](#_Toc101437292)

[Figure S29: Reoperation, acute surgery subgroup analysis 56](#_Toc101437293)

[Figure S30: Atelectasis, meta-analysis 57](#_Toc101437294)

[Figure S31: Pneumonia, meta-analysis 58](#_Toc101437295)

[Figure S32: Atelectasis, abdominal surgery subgroup analysis 59](#_Toc101437296)

[Figure S33: Pneumonia, abdominal surgery subgroup analysis 60](#_Toc101437297)

[Figure S34: Myocardial injury/myocardial infarction, meta-analysis 61](#_Toc101437298)

[Figure S35: Myocardial injury/myocardial infarction, abdominal surgery subgroup analysis 62](#_Toc101437299)

[PRISMA CHECKLIST 63](#_Toc101437300)

[REFERENCES 66](#_Toc101437301)

# **SUPPLEMENTAL METHODS**

## **Outcomes**

*Mortality*

Mortality was defined as all-cause mortality. We considered the timeframes in-hospital, during study period, 7-day, 15-day, and 30-day mortality as comparable, and they were grouped as short-term mortality. Mortality after 30 days was considered as long-term mortality. Long-term mortality ranged from 180-days to 4 years across the five included trials. We also included an analysis of overall mortality considering all timeframes. If a trial reported more than one timeframe, we used the time closest to 30 days.

*Hospital length of stay*

The respective authors’ definitions of hospital length of stay were accepted without requirement of further specification. Length of stay was reported as both median with quartiles and as mean with standard deviation (SD). Medians and SD were estimated from medians and quartiles as described in the main manuscript. One trial reported length of stay as mean with range, and this trial was excluded as this does not allow for estimation of mean and SD. Two trials in patients undergoing ambulatory gynecological laparoscopy were excluded as the length of stay was less than 6 hours in the intervention as well as the control group.

*Surgical site infection*

The majority of the trials defined surgical site infection according to the Centers for Disease Control and Prevention criteria^1^ or referring to ASEPSIS score^2^ (most studies included a score $\geq$ 20 as evidence of wound infection). Only a few trials used definitions based on non-standardized measurements. Only one trial did not report their definition of surgical site infection. The timeframe for data collection on this complication ranged from first 7 postoperative days to 12 months postoperatively. The majority, however, reported data from within the first 30 postoperative days.

*Anastomotic leakage*

Most of the included trials reporting data on anastomotic leakage did not specify a definition. One trial defined it as requiring antibiotics ± surgery. All the studies reporting data on anastomotic leakage were included.

*Wound dehiscence*

Wound dehiscence was defined as requiring secondary suture of the fascia combined with identification of ICD-9 codes or as requiring surgical closure of the cutaneous or subcutaneous tissue or the fascia and muscular plane during the early postoperative period. Only one trial did not define this postoperative complication.

*Reoperation*

This outcome was not defined across all trials, although the majority specified the outcome as patients requiring ‘wound exploration’ or ‘reoperation’.

*Atelectasis*

Atelectasis was defined based on radiological findings on postoperative x-rays or CT-scans for most included trials. Three of eight included trials provided no explicit definition hereof.

*Pneumonia*

The authors’ criteria for defining pneumonia, including trials using guideline definitions, were only explicitly defined in 3 of 7 included trials. For the remainder of trials, pneumonia was either defined based on clinical and radiological signs or not defined at all. Guideline definitions included CDC^3^ and ARISCAT^4^ criteria.

*Myocardial injury/infarction*

This outcome was reported quite heterogeneously in the included trials and was therefore included as “myocardial injury/infarction” in this review. Four studies used blood samples for troponin T/I, two studies used definitions pertinent to “acute coronary syndrome”, and one study used ICD-10 codes to identify acute coronary syndrome, unstable angina pectoris, and myocardial infarction as a composite outcome. Thus, both cases of type I infarction (i.e., infarction due to coronary thrombus formation) and type II infarction (i.e., postoperative rise in troponins) are included in this outcome.

## **Risk of bias assessment**

Risk of bias was assessed using version 2 of the Cochrane Risk-of-Bias tool for individually-randomized parallel-group trials.^5^ Results for risk of bias are presented in Table S4. General considerations related to this specific review are provided below.

*Risk of bias arising from the randomization process*

Three elements are considered within this domain: random allocation sequence, allocation concealment, and baseline imbalances. In general, if randomization was described and there was no indication of loss of allocation concealment, we categorized the trial as low risk of bias – this included trials with no clear description of allocation concealment as we then assumed it to be present. If the randomization process was not described at all, we generally considered trials to be in intermediate risk of bias.

*Risk of bias due to deviations from the intended interventions*

This domain focuses on whether participants received the intended intervention without other differences in care. As such, the domain primarily focuses on blinding of participants and the clinical team. It is difficult to blind the clinician who provides the intervention, and for much of the treatment data it is infeasible to decipher whether differences result from the intervention or non-protocol treatments. We therefore decided to judge all trials as at least intermediate risk of bias.

The difference between the effect of assignment to an intervention and the effect of adherence to an intervention was difficult to assess and often not described in the trials. Although we were technically interested in the effect of adherence to the intervention, the distinction between the two is probably minimal and therefore not considered further. Since appropriate per-protocol analyses were rarely performed, we – when possible – included results from (modified) intention-to-treat analyses.

*Risk of bias due to missing outcome data*

For most trials, outcomes were only assessed in-hospital, and the amount of missing data was low. These trials were therefore classified as having a low risk of bias. In a few trials, loss to follow-up was more substantial. When this loss to follow-up was moderate and equal among groups, the risk of bias was assessed as intermediate; if the loss to follow-up was more substantial and different between groups, the risk of bias was classified as high.

*Risk of bias in measurement of the outcome*

This domain includes multiple elements including whether the method of measuring the outcome was appropriate and similar between groups, whether outcome assessors were aware of the intervention, and whether this knowledge could have influenced the measurement of the outcome.

For most trials, the methods of measuring the outcomes were described very limited. Survival and hospital length of stay were considered objective outcomes, why knowledge of the intervention is unlikely to influence measurement of the outcome, and risk of bias was therefore considered low. For postoperative complications, the risk of bias was classified as low if the outcomes assessors were blinded and intermediate if they were not. We considered it unlikely that this non-blinding would have a major influence on the measurement of the outcome.

*Risk of bias in selection of the reported result*

If the trials reported results consistent with a protocol or trial registration, this domain was classified as low risk of bias. If there was no protocol or trial registration, the trial was classified as intermediate risk of bias. Outcome-relevant discrepancies between the manuscript and the protocol or trial registration were classified as intermediate or high based on whether or not it was selected from multiple outcome measurements or analyses of the data.

*Overall risk of bias*

The overall risk of bias was generally based on the highest risk reported within an individual domain.

## **Search for ongoing trials**

The International Clinical Trials Registry Platform (ICTRP) was searched on April 5, 2021, and on June 28, 2021. The searches yielded 4034 records, of which 25 records were ongoing randomized controlled trials comparing oxygenation targets during general anesthesia (Table S1).

To optimize sensitivity, we performed an additional search for the term *general anesthesia* (filters: recruiting, not yet recruiting, enrolling by invitation, active but not recruiting, and interventional study type) on ClinicalTrials.Gov on April 5, 2021. Of 311 records, no additional randomized controlled trials were identified.

We did not consider records for which 1) the recruitment status was unknown, 2) the trial status was marked as unknown for more than 5 years, 3) the trial was marked as completed for more than 5 years, and/or 4) the last update was posted over 5 years ago.

Search at ICTRP on April 5, 2021:

*anesthesia AND hemodynamic OR anesthesia AND respiratory OR surgery AND hemodynamic OR surgery AND respiratory*

Search at ICTRP on June 28, 2021:

*((anesthesia OR anaesthesia OR surgery OR surgical) AND (arterial blood pressure OR heart rate OR cardiac output OR stroke volume OR pulse pressure OR goal directed therapy OR goal-directed therapy OR oxygen OR carbon dioxide OR tidal volume OR respiratory rate OR PEEP OR recruitment maneuver OR lung protective ventilation))*

## **Updated search for studies**

An updated search for the term *oxygen* was performed in PubMed and Embase on March 22, 2022, to identify any trials published after the search on March 8, 2021. The search strategies are provided below.

PubMed:

*(Anesthesia, General[Mesh] OR universal anesthesia[Title/Abstract] OR universal anaesthesia[Title/Abstract] OR general anesthesia[Title/Abstract] OR general anaesthesia[Title/Abstract] OR General Surgery[Mesh] OR surgery[Title/Abstract] OR surgical[Title/Abstract] OR perioperative[Title/Abstract] OR “peri-operative”[Title/Abstract] OR intraoperative[Title/Abstract] OR “intra-operative”[Title/Abstract]) AND (oxygen[Title/Abstract]) AND (randomized controlled trial[Publication Type] OR controlled clinical trial[Publication Type] OR randomized[Title/Abstract] OR placebo[Title/Abstract] OR clinical trials as topic[Mesh:noexp] OR randomly[Title/Abstract] OR trial[Title]) NOT (animals[Mesh] NOT humans [Mesh]) AND (English[Language])*

EMBASE:

*('anesthesia'/exp OR 'universal anesthesia':ti,ab OR 'universal anaesthesia':ti,ab OR 'general anesthesia':ti,ab OR 'general anaesthesia':ti,ab OR 'surgery':ti,ab OR 'surgical':ti,ab OR 'perioperative':ti,ab OR 'intraoperative':ti,ab) AND ('oxygen':ti,ab OR 'arterial oxygen pressure':ti,ab) AND ('randomized controlled trial'/exp OR 'controlled clinical trial'/exp OR randomly:ti,ab OR randomized:ti,ab OR placebo:ti,ab OR trial:ti,ab) NOT ('animal'/exp NOT 'human'/exp) AND ('article'/it OR 'article in press'/it) AND [english]/lim*

## **GRADE evaluation**

The GRADE methodology was used to evaluate the overall certainty in the evidence for a given intervention and outcome. The following domains were considered.

*Risk of bias*

Risk of bias was rated as “serious” for all interventions and outcomes since almost all of the individual trials were assessed as having an intermediate risk of bias.

*Inconsistency*

Inconsistency was evaluated with the I^2^ statistic as well as visual inspection of the forest plot. As a guide, inconsistency was considered “serious” if the I^2^ statistic was > 30%, but this also depended on the distribution of the individual trials.

*Indirectness*

Given that all the trials met our predefined criteria for inclusion and therefore represent the patient population of interest, indirectness was considered “not serious”. However, if the interest is in specific patient populations defined by patient characteristics or surgery type, it could be considered to assess indirectness as “serious”.

*Imprecision*

In determining whether a given comparison and outcome should be downgraded for imprecision, we considered both the width of the confidence interval and the sample size (in that order). For binary outcomes, we mainly considered the effect on the relative scale (i.e., odds ratio), but also evaluated the effect on the absolute scale (i.e., risk difference). If the confidence interval was very wide (i.e., < 0.50 and > 2.00 for the odds ratio) and included both potential benefit and harm, we considered imprecision to be “very serious”. If the confidence was less wide and included potential benefit and no clear effect or harm, imprecision was considered “serious”. If the confidence interval only included clear benefit or harm or was narrow around no effect (i.e., within 0.80 to 1.20 for the odds ratio), we considered the sample size in relation to the optimal information size.^6^ For length of stay, a narrow confidence interval was considered within -0.25 to 0.25 days.

The optimal information size is equivalent to the required sample size of a single, adequately powered trial. It is challenging to determine a general optimal information size. However, considering an absolute risk reduction of 1%, the optimal information size would be 6,206 for a control group outcome proportion of 2% and 39,494 for a control group outcome proportion of 11%. If a 5% absolute risk reduction was considered, the optimal information size would be 1,164 for a control group outcome proportion of 10% and 2,424 for 20%. These calculations are based on a chi-squared test, an alpha of 5%, and 90% power. Based on these considerations and previous suggestions^6^, we considered imprecision to be “serious” if the sample size was less than 2,000 patients. If the sample size was less than 100 patients, we considered imprecision to be “very serious”. For consistency, we used the same sample sizes for length of stay.

*Others*

In this domain, multiple aspects are considered, including publication bias, the size of the effect, the direction of potential bias, and dose response gradients. The last two were not of relevance in the current review. Given the small number of trials, it was generally not feasible to evaluate for publication bias. We considered a large effect to be < 0.50 or > 2.00 on the odds ratio scale. For hospital length of stay, we considered a large effect to be > 2 days.

# **TABLES**

| **Table S1: Registered randomized trials comparing oxygen targets** | | | | | | | | | |
| --- | --- | --- | --- | --- | --- | --- | --- | --- | --- |
| Title | ID | Country | Year registered | Estimated completion | Comparator group | Comparator group | Comparator group | Patients | Status |
| Effects of inspiring oxygen concentration on pulmonary oxygenation and respiratory mechanics in morbidly obese patients undergoing laparoscopic bariatric surgery | ChiCTR1900020619 | China | 2019 | 12/2019 | FiO_2_ 40% | FiO_2_ 60% | FiO_2_ 80% | 60 | Not recruiting |
| The effect of perioperative oxygen supplementation to reduce the incidence of surgical site infections in patients undergoing elective major abdominal surgeries | CTRI/2021/02/031436 | India | 2021 | 01/2022 | FiO_2_ and 70% nitrous oxide | FiO_2_ and 20% nitrous oxide | NA | 50 | Not recruiting |
| Perioperative respiratory care and outcomes for patients undergoing high risk abdominal surgery (PENGUIN) | NCT04256798 | South Africa | 2020 | 06/2024 | Mouthwash and/or liberal oxygen | Mouthwash and/or restrictive oxygen | NA | 12,942 | Recruiting |
| Immunological effect of intraoperative fraction of inspired oxygen in patients undergoing major abdominal cancer surgery | NCT04772794 | Egypt | 2021 | 05/2022 | FiO_2_ 80% | FiO_2_ 30% | NA | 20 | Recruiting |
| Pulmonary complications and clinical outcomes using ultrasound according to the inspired oxygen concentration during general anesthesia: multi-center, randomized controlled trials | KCT0005872 | South Korea | 2021 | 12/2021 | FiO_2_ 100% | FiO_2_ 40% | NA | 320 | Not recruiting |
| Effects of different oxygen concentrations on air embolization in hysteroscopic surgery: a randomized controlled trial | ChiCTR2000033202 | China | 2020 | 09/2020 | FiO_2_ 30% | FiO_2_ 50% | FIO2 100% | 120 | Not recruiting |
| Effect of intraoperative oxygen use on cerebral blood flow in adult patients under general anaesthesia: a randomized controlled trial | CTRI/2020/03/024295 | India | 2020 | 04/2020 | FiO_2_ 30% | FiO_2_ 80% | NA | 56 | Not recruiting |
| Effect of different fraction of inspired oxygen on ventilator-induced lung injury in patients during prolonged general anesthesia | ChiCTR2000029075 | China | 2020 | 02/2021 | FiO_2_ 30% | FiO_2_ 50% | FiO2 80% | 90 | Recruiting |
| Is peri-operative hyperoxemia a risk factor for postoperative complications? A randomised, prospective study in patients undergoing vascular surgery | NCT02562781 | Sweden | 2015 | 01/2021 | FiO_2_ > 50% and SpO_2_ of 98-100% | Air or lowest possible inspired concentration of oxygen to maintain SpO_2_ > 90% | NA | 184 | Completed |
| Comparison of oxygen exposure amount between oxygen reserve index based FiO2 titration and conventional FiO2 titration in thoracic surgery | KCT0004782 | South Korea | 2020 | 02/2021 | Oxygen fraction with the aim of ORI 0.21 | Standard of care | NA | 140 | Not recruiting |
| Efficacy of oxygen reserve index (ORI) to reduce hyperoxia in major abdominal surgery | NCT04211246 | South Korea | 2019 | 12/2021 | FiO_2_ titrated by SpO2 | FiO_2_ titrated by SpO2 and ORI | NA | 64 | Recruiting |
| Increasing FiO2 influences accuracy of fick-based assessments of cardiac output in cardiac surgery patients | NCT03970980 | Taiwan | 2019 | 01/2017 | FiO_2_ >90% | FiO_2_ <70% | NA | 24 | Completed |
| A study on the effects of hyperbaric oxygen therapy on preventing surgical site infection in orofacial surgery - a study on the effects of hyperbaric oxygen therapy on preventing surgical site infection in orofacial surgery | JPRN-UMIN000035786 | Japan | 2019 | 09/2022 | FiO_2_ 80% | FiO_2_ 30% | NA | 300 | Completed |
| Perioperative utilisation of supplemental oxygen | NCT03552627 | USA | 2018 | 01/2022 | FiO_2_ 80% | FiO_2_ 55% | FiO_2_ 30% | 39 | Recruiting |
| Efficacy of high oxygen supply in perioperative period of thyroid surgery to prevent postoperative laryngopharyngeal symptoms, recurrent laryngeal nerve palsy, and parathyroid dysfunction: a double blind randomized controlled trial | ChiCTR-POR-17012765 | China | 2017 | 10/2018 | FiO_2_ 80% | FiO_2_ 30% | NA | 600 | Not recruiting |
| Supplemental perioperative oxygen to reduce surgical site infection after high energy fracture surgery (OXYGEN study) | NCT01798810 | USA | 2013 | 06/2021 | FiO_2_ 80% | FiO_2_ 30% | NA | 1,000 | Not recruiting |
| Effect of different inhaled oxygen concentration on cerebral oxygen saturation and delirium after abdominal surgery | ChiCTR1900022371 | China | 2019 | 06/2020 | FiO_2_ 40% | FiO_2_ 60% | FiO2 80% | 480 | Recruiting |
| A multi-centre, patient and assessor-blinded, parallel groups, randomised controlled trial comparing restricted and liberal oxygen therapy with standard oxygen therapy in patients having major surgery | ACTRN12619000115134 | New Zealand | 2019 | 06/2022 | Minimal FiO_2_ to achieve an SpO2 greater than or equal to 93% | FiO_2_ 80% | FiO2 between 0.4 and 0.6 | 210 | Recruiting |
| Effect of different fraction of inspired oxygen concentrations on cerebral oxygen saturation and postoperative cognitive function in elderly patients undergoing spinal surgery | ChiCTR-IOR-17013775 | China | 2017 | 10/2018 | FiO_2_ 30% | FiO_2_ 60% | FiO2 80% | 60 | Recruiting |
| The effect of supplemental oxygen during the procedure on postoperative nausea and vomiting in patients undergoing cataract surgery | IRCT2016020126300N1 | Iran | 2016 | 09/2016 | FiO_2_ 30% | FiO_2_ 80% | NA | 200 | Completed |
| Hyperoxia and antioxidants during major non-cardiac surgery and risk of cardiovascular complications, a blinded 2x2 factorial randomised clinical trial | NCT03494387 | Denmark | 2018 | 03/2020 | FiO_2_ 80% | FiO_2_ 30% | NA | 600 | Completed |
| Is high oxygen concentration a risk factor for postoperative complications? A prospective, randomized, single blinded study in elderly patients undergoing vascular surgery | EUCTR2016-001584-36-SE | Sweden | 2016 | NR | FiO_2_ <30% | FiO_2_ >50% | NA | 200 | Ongoing |
| Cardiac complications associated to perioperative hyperoxia in elective colorectal surgery | EUCTR2018-001917-34-ES | Spain | 2018 | NR | FiO_2_ 80% | FiO_2_ 40% | NA | 400 | Ongoing |
| Reduction of surgical wound infection breathing high concentrations of oxygen during anesthesia | EUCTR2016-002936-34-ES | Spain | 2016 | NR | FiO_2_ 80% | FiO_2_ 30% | NA | 756 | Completed |
| The influence of 100% oxygen inhaled under hyperbaric conditions on traumatic injury of the spinal cord. | EUCTR2018-004679-11-AT | Austria | 2020 | NR | FiO_2_ 100% | NR | NA | 100 | Ongoing |

| **Table S2: Patient and surgical characteristics in included manuscripts** | | | | | | | | | |
| --- | --- | --- | --- | --- | --- | --- | --- | --- | --- |
| Study | Age^a^ | Sex (% male) | BMI^a^ | ASA I+II (%) | Abdominal surgery (%) | Laparoscopic surgery (%) | Acute surgery (%) | Duration of surgery (min)^a^ | Duration of anesthesia (min)^a^ |
| Kotani, 2000^7^ | 49 | 60 | NR | 100 | 0 | NR | 0 | NR | 495 |
| Greif, 2000^8^ | 57 | 56 | 25 | 84 | 100 | 0 | NR | 186 | NR |
| Purhonen, 2002^9^ | 37 | 0 | 24 | NR | 0 | 100 | 0 | 31 | 43 |
| Pryor, 2004^10^ | 56 | 43 | 26 | 78 | 83 | 0 | NR | 221 | NR |
| Mayzler, 2005^11^ | 68 | 58 | 26 | NR | 100 | NR | 0 | 138 | NR |
| Belda, 2005^12^ | 63 | 56 | 27 | 73 | 100 | 0 | 0 | 160 | NR |
| Myles, 2007^13^ | 55 | 52 | NR | 76 | 46 | NR | 4 | 198 | 222 |
| Meyhoff, 2009^14^ | 64 | 42 | 25 | 81 | 81 | 0 | 28 | 130 | 193 |
| McKeen, 2009^15^ | 36 | 0 | 26 | 100 | 0 | 100 | 0 | 22 | 44 |
| Bickel, 2011^16^ | 28 | 73 | NR | NR | 100 | 0 | 100 | 33 | NR |
| Thibon, 2012^17^ | 52 | 10 | 25 | 96 | 20 | 27 | NR | 87 | NR |
| Staehr, 2012^18^ | 62 | 0 | 25 | 83 | 0 | 0 | 0 | NR | 167 |
| Meyhoff, 2012^19^ | 64 | 42 | 25 | 81 | NR | 0 | 27 | 130 | NR |
| Stall, 2013^20^ | 42 | 69 | 28 | NR | 0 | 0 | 100 | 230 | NR |
| Chen, 2013^21^ | 61 | 58 | NR | NR | 100 | 0 | 0 | NR | 192 |
| Meyhoff, 2014^22^ | 64 | 43 | 25 | 81 | NR | 0 | 28 | 129 | NR |
| Kurz, 2015^23^ | 53 | 50 | 27 | 74 | 100 | 32 | 0 | 210 | NR |
| Wasnik, 2015^24^ | 28 | 20 | NR | NR | 100 | 0 | 100 | 61 | NR |
| Fonnes, 2016^25^ | 64 | 42 | 25 | 81 | 100 | 0 | 28 | 130 | NR |
| Chiang, 2017^26^ | 70 | 66 | 27 | NR | 0 | 0 | 0 | 229 | NR |
| Kurz, 2017^27^ | 52 | 48 | 26 | 32 | 100 | 27 | NR | 240 | NR |
| Mayank, 2018^28^ | 55 | 62 | 21 | 59 | 100 | NR | 0 | 221 | NR |
| Kongebro, 2018^29^ | 64 | 42 | 25 | 81 | 100 | 0 | 29 | NR | 193 |
| Alvandipour, 2018^30^ | 59 | 25 | 26 | NR | 100 | 0 | 31 | NR | NR |
| Ruetzler, 2019^31^ | 52 | 48 | 26 | 32 | 100 | 27 | NR | 263 | NR |
| Cohen, 2019^32^ | 52 | 52 | 27 | 33 | 100 | 2 | 3 | 240 | NR |
| Ferrando, 2019^33^ | 64 | 38 | 26 | 66 | 82 | 50 | NR | NR | NR |
| Li, 2020^34^ | 54 | 50 | 23 | 86 | 100 | 27 | 0 | 195 | 260 |
| Jiang, 2021^35^ | 54 | 49 | 27 | 30 | 100 | 28 | NR | 252 | NR |
| Lin, 2021^36^ | 71 | 54 | 23 | 88 | 100 | 100 | NR | 188 | 224 |
| Park, 2021^37^ | 63 | 69 | 24 | 93 | 100 | 83 | 0 | NR | NR |
| Reiterer, 2021^38^ | 74 | 67 | 26 | 29 | 46 | NR | 0 | 195 | 261 |
| Holse, 2022^39^ | 72 | 59 | 27 | 46 | 51 | 36 | NR | 132 | 205 |
| *BMI: body mass index, ASA: American Society of Anaesthesiologists classification, NR: not reported.* a: Mean or median | | | | | | | | | |

| **Table S3: Reported outcomes in the included manuscripts^a^** | | | | | | | | | | | | | | | | | | | |
| --- | --- | --- | --- | --- | --- | --- | --- | --- | --- | --- | --- | --- | --- | --- | --- | --- | --- | --- | --- |
| Trial | Mortality | LOS^b^ | Atelectasis | ARDS | Respiratory failure | Extubation failure/reintubation | Pleural effusion | Pneumonia | Composite pulmonary | MI | Arrhythmias | Composite cardiac | SSI | Neurological complications | Renal failure | Sepsis | Anastomotic leakage | Wound dehiscence | Reoperation |
| Kotani, 2000^7^ |  |  | X |  |  |  |  | X |  |  |  |  |  |  |  |  |  |  |  |
| Greif, 2000^8^ | X | X | X^c^ |  |  |  |  |  |  |  |  |  | X |  |  |  |  |  |  |
| Purhonen, 2002^9^ |  | X |  |  |  |  |  |  |  |  |  |  |  |  |  |  |  |  |  |
| Pryor, 2004^10^ | X | X |  |  |  |  |  |  |  |  |  |  | X |  |  |  |  |  | X |
| Mayzler, 2005^11^ |  |  |  |  |  |  |  |  |  |  |  |  | X |  |  |  | X |  |  |
| Belda, 2005^12^ | X | X |  |  |  |  |  |  |  |  |  |  | X |  |  |  |  |  |  |
| Myles, 2007^13^ | X | X | X |  |  |  |  | X |  | X |  |  | X | X |  |  |  |  |  |
| Meyhoff, 2009^14^ | X | X | X |  |  |  |  | X |  |  |  | X | X |  |  | X | X |  | X |
| McKeen, 2009^15^ |  | X |  |  |  |  |  |  |  |  |  |  |  |  |  |  |  |  |  |
| Bickel, 2011^16^ |  | X |  |  |  |  |  |  |  |  |  |  | X |  |  |  |  |  | X |
| Thibon, 2012^17^ |  |  |  |  |  |  |  |  |  |  |  |  | X |  |  |  |  |  |  |
| Staehr, 2012^18^ |  |  | X |  |  |  |  |  |  |  |  |  |  |  |  |  |  |  |  |
| Meyhoff, 2012^19^ | X |  |  |  |  |  |  |  |  |  |  |  |  |  |  |  |  |  |  |
| Stall, 2013^20^ |  | X |  |  |  |  |  |  |  |  |  |  | X |  |  |  |  |  |  |
| Chen, 2013^21^ | X | X |  |  |  |  |  | X |  | X |  |  | X |  |  |  |  |  |  |
| Meyhoff, 2014^22^ | X |  |  |  |  |  |  |  |  |  |  |  |  |  |  |  |  |  |  |
| Kurz, 2015^23^ | X | X |  |  |  |  |  |  |  |  |  |  | X |  |  |  |  |  |  |
| Wasnik, 2015^24^ |  | X |  |  |  |  |  |  |  |  |  |  | X |  |  |  |  |  |  |
| Fonnes, 2016^25^ |  |  |  |  |  |  |  |  |  | X | X |  |  |  |  |  |  |  |  |
| Chiang, 2017^26^ | X |  |  |  |  |  |  | X |  | X^d^ |  |  | X |  |  |  |  | X |  |
| Kurz, 2017^27^ | X |  |  |  |  |  |  |  |  |  |  |  | X |  |  | X | X | X |  |
| Mayank, 2018^28^ | X | X |  |  |  |  |  |  |  |  |  |  | X |  |  |  | X |  |  |
| Kongebro, 2018^29^ | X |  |  |  |  |  |  |  |  |  |  |  |  | X |  |  |  |  |  |
| Alvandipour, 2018^30^ |  | X | X |  |  |  |  |  |  |  |  |  | X |  |  |  | X |  |  |
| Ruetzler, 2019^31^ | X | X |  |  |  |  |  |  |  | X |  |  |  |  | X |  |  |  |  |
| Cohen, 2019^32^ | X |  | X |  |  | X |  | X | X |  |  |  |  |  |  |  |  |  |  |
| Ferrando, 2019^33^ | X | X | X |  |  |  |  |  | X |  |  | X | X |  |  |  |  |  |  |
| Li, 2020^34^ | X | X | X |  |  | X |  | X |  | X |  |  | X |  | X | X |  |  |  |
| Jiang, 2021^35^ | X |  |  |  |  |  |  |  |  |  |  |  |  |  |  |  |  |  |  |
| Lin, 2021^36^ | X | X | X |  |  |  |  | X |  | X |  |  | X |  |  |  |  |  |  |
| Park, 2021^37^ |  | X | X | X |  | X |  | X |  |  | X |  | X |  |  |  |  |  | X |
| Reiterer, 2021^38^ | X |  |  |  | X |  |  |  |  | X |  |  |  |  |  |  |  |  | X |
| Holse, 2022^39^ | X |  |  |  | X |  |  | X |  | X |  |  | X |  | X | X |  |  |  |
| *LOS: length of stay, ARDS: acute respiratory distress syndrome, MI: myocardial infarction, SSI: surgical site infection,*  a: Raw data provided in the separate forest plots for meta-analysis  b: Hospital/post-anaesthesia care unit/intensive care unit  c: Reported in subanalysis^40^  d: Acute coronary syndrome | | | | | | | | | | | | | | | | | | | |

| **Table S4: Bias assessment of included manuscripts** | | | | | | |
| --- | --- | --- | --- | --- | --- | --- |
| Study | Randomization | Adherence to intervention^b^ | Missing outcome data | Measurement of the outcome | Selective reporting^d^ | Overall |
| Kotani, 2000^7^ | Low | Intermediate | Low | Intermediate | Intermediate | Intermediate |
| Greif, 2000^8^ | Low | Intermediate | Low | Low | Intermediate | Intermediate |
| Purhonen, 2002^9^ | Low | Intermediate | Low | Low | Intermediate | Intermediate |
| Pryor, 2004^10^ | Low | Intermediate | Low | Low | Intermediate | Intermediate |
| Mayzler, 2005^11^ | Low | Intermediate | Low | Low | Intermediate | Intermediate |
| Belda, 2005^12^ | Low | Intermediate | Low | Low | Intermediate | Intermediate |
| Myles, 2007^13^ | Low | Intermediate | Low | Low | Intermediate | Intermediate |
| Meyhoff, 2009^14^ | Low | Intermediate | Low | Low | Low | Intermediate |
| McKeen, 2009^15^ | Low | Intermediate | Low | Low | Intermediate | Intermediate |
| Bickel, 2011^16^ | Low | Intermediate | Low | Low | Intermediate | Intermediate |
| Thibon, 2012^17^ | Low | Intermediate | Low | Low | Intermediate | Intermediate |
| Staehr, 2012^18^ | Low | Intermediate | Low | Low | Intermediate | Intermediate |
| Meyhoff, 2012^19^ | Low | Intermediate | Low | Low | Intermediate | Intermediate |
| Stall, 2013^20^ | Low | Intermediate | Low | Low | Intermediate | Intermediate |
| Chen, 2013^21^ | Low | Intermediate | Low | Low | Low | Intermediate |
| Meyhoff, 2014^22^ | Low | Intermediate | Low | Low | Intermediate | Intermediate |
| Kurz, 2015^23^ | Low | Intermediate | Low | Low | Intermediate | Intermediate |
| Wasnik, 2015^24^ | Intermediate^e^ | Intermediate | Intermediate^e^ | Low | Low | Intermediate |
| Fonnes, 2016^25^ | Low | Intermediate | Low | Low | Intermediate | Intermediate |
| Chiang, 2017^26^ | Low | Intermediate | Low | Low | Intermediate | Intermediate |
| Kurz, 2017^27^ | Intermediate^a^ | Intermediate | Low | Low | Low | Intermediate |
| Mayank, 2018^28^ | Low | Intermediate | Low | Low | Low | Intermediate |
| Kongebro, 2018^29^ | Low | Intermediate | Low | Low | Intermediate | Intermediate |
| Alvandipour, 2018^30^ | Low | Intermediate | Low | Low | Intermediate | Intermediate |
| Ruetzler, 2019^31^ | Intermediate^a^ | Intermediate | Intermediate^c^ | Low | Intermediate | Intermediate |
| Cohen, 2019^32^ | Intermediate^a^ | Intermediate | Low | Low | Intermediate | Intermediate |
| Ferrando, 2019^33^ | Low | Intermediate | Low | Low | Low | Intermediate |
| Li, 2020^34^ | Low | Intermediate | Low | Intermediate | Low | Intermediate |
| Jiang, 2021^35^ | Intermediate^a^ | Intermediate | Low | Low | Low | Intermediate |
| Lin, 2021^36^ | Low | Intermediate | Intermediate^c^ | Intermediate | Intermediate | Intermediate |
| Park, 2021^37^ | Low | Intermediate | Low | Low | Intermediate | Intermediate |
| Reiterer, 2021^38^ | Low | Intermediate | Low | Low | Low | Intermediate |
| Holse, 2022^39^ | Low | Intermediate | Low | Intermediate | Low | Intermediate |
| a: No allocation sequence concealment  b: All studies intermediate risk of bias due to anaesthesiologist not being blinded  c: Many patients excluded due to missing data  d: Intermediate due to no published protocol  e: Not described | | | | | | |

| **Table S5. Meta regression analyses** | | | | | |
| --- | --- | --- | --- | --- | --- |
| Outcomes and moderators | Number of  studies | Intercept | β-coefficients  (95% CI) | Ratio of geometric mean ORs* (95% CI) | P value |
| **Mortality** |  |  |  |  |  |
| Year of patient inclusion (per year) | 12 | -149.1 | 0.074  (0.023, 0.126) | 1.08  (1.02, 1.13) | 0.005 |
| Mortality in the control group (per 1%) | 12 | 0.307 | -0.066  (-0.266, 0.133) | 0.94 (0.77, 1.14) | 0.52 |
| Sample size (per 100 subjects) | 12 | -0.083 | 0.0125  (-0.004, 0.029) | 1.01 (1.00, 1.03) | 0.15 |
| **Hospital length of stay** |  |  |  |  |  |
| Year of patient inclusion (per year) | 14 | 46.92 | -0.023  (-0.064, 0.018) | NA | 0.27 |
| Mortality in the control group (per 1%) | 12 | -0.056 | -0.228  (-0.985, 0.528) | NA | 0.55 |
| Length of stay in control group (per day) | 14 | 0.328 | -0.027  (-0.122, 0.068) | NA | 0.57 |
| Duration of surgery (per minute) | 10 | -0.653 | 0.005  (-0.001, 0.011) | NA | 0.09 |
| Sample size (per 100 subjects) | 14 | 0.183 | -0.031  (-0.057, -0.004) | NA | 0.03 |
| **Surgical site infection** |  |  |  |  |  |
| Year of patient inclusion (per year) | 17 | -35.84 | 0.018  (-0.004, 0.040) | 1.02 (1.00, 1.04) | 0.11 |
| Mortality in the control group (per 1%) | 12 | -0.053 | -0.043  (-0.137, 0.051) | 0.96  (0.87, 1.05) | 0.37 |
| Length of stay in control group (per day) | 13 | -0.454 | 0.024  (-0.012, 0.061) | 1.02 (0.99, 1.06) | 0.19 |
| Duration of surgery (per minute) | 14 | -0.531 | 0.002  (-0.001, 0.005) | 1.00 (1.00, 1.01) | 0.11 |
| Sample size (per 100 subjects) | 17 | -0.232 | 0.004  (-0.002, 0.010) | 1.00 (1.00, 1.01) | 0.16 |
| *CI: confidence interval, OR: odds ratio* | | | | | |

* Not reported for hospital length of stay as the β-coefficients represents the mean difference in effect size per each each unit change in the moderator variable

| **Table S6: Sensitivity analyses** | | | | | |
| --- | --- | --- | --- | --- | --- |
| Analysis | Number of trials | Number of patients | | Heterogeneity (I^2^ [%]) | Effect estimate (95% CI) |
|  |  | Intervention | Control |  |  |
| ***Short-term mortality*** | | | | | |
| Primary analysis | 12 | 6183 | 6152 | 38 | 1.27 (0.90; 1.79) |
| Excluding trials with FiO_2_ ≠ 80% or 30% | 10 | 6056 | 6052 | 40 | 1.29 (0.91; 1.83) |
| ***Long-term mortality*** | | | | | |
| Primary analysis | 6 | 3283 | 3249 | 66 | 1.04 (0.90; 1.21) |
| Excluding trials with FiO_2_ ≠ 80% or 30% | 4 | 2819 | 2792 | 64 | 1.08 (0.93; 1.26) |
| ***Hospital length of stay*** | | | | | |
| Primary analysis | 17 | 4529 | 4535 | 70 | 0.03  (-0.25, 0.30) |
| Excluding trials with FiO_2_ ≠ 80% or 30% | 12 | 3853 | 3864 | 66 | -0.14  (-0.53, 0.25) |
| ***Surgical site infection*** | | | | | |
| Primary analysis | 21 | 7168 | 7100 | 38 | 0.91 (0.81; 1.02) |
| Excluding trials with FiO_2_ ≠ 80% or 30% | 16 | 6765 | 6701 | 35 | 0.88 (0.78; 0.99) |
| ***Anastomotic leakage*** | | | | | |
| Primary analysis | 5 | 3687 | 3660 | 0 | 0.78 (0.54; 1.14) |
| Excluding trials with FiO_2_ ≠ 80% or 30% | 4 | 3640 | 3613 | 3 | 0.79 (0.54; 1.16) |
| ***Reoperation*** | | | | | |
| Primary analysis | 5 | 1083 | 105 | 47 | 1.09 (0.84; 1.43) |
| Excluding trials with FiO_2_ ≠ 80% or 30% | 3 | 916 | 938 | 0 | 1.04 (0.79; 1.3643) |
| ***Atelectasis*** | | | | | |
| Primary analysis | 8 | 4826 | 4766 | 61 | 0.90 (0.78, 1.04) |
| Excluding trials with FiO_2_ ≠ 80% or 30% | 7 | 4796 | 4736 | 65 | 0.96 (0.74, 1.24) |
| ***Pneumonia*** | | | | | |
| Primary analysis | 7 | 4457 | 4405 | 52 | 0.96 (0.74, 1.24) |
| Excluding trials with FiO_2_ ≠ 80% or 30% | 6 | 4427 | 4375 | 52 | 0.96 (0.74, 1.24) |
| *CI: confidence interval, FiO_2_: Fraction of inspired oxygen* | | | | | |

| **Table S7: GRADE assessment** | | | | | | | | | | | | |
| --- | --- | --- | --- | --- | --- | --- | --- | --- | --- | --- | --- | --- |
|  | **Certainty assessment** | | | | | | | **Number of patients** | | **Effect** | | **Certainty** |
| **Outcome** | **№ of studies** | **Study design** | **Risk of bias** | **Inconsistency** | **Indirectness** | **Imprecision** | **Other considerations** | **FiO_2_ 80%** | **FiO_2_ 30%** | **Odds ratio (95% CI)** | **Absolute (95% CI)** |  |
| Short-term mortality | 12 | Randomised trials | Not serious | Serious ^a^ | Not serious | Serious ^b^ | None | 73/6183 (1.2%) | 58/6152 (0.9%) | **OR 1.27**  (0.90 to 1.79) | **3 more per 1,000**  (from 1 fewer to 7 more) | ⨁⨁◯◯ LOW |
| Long-term mortality | 6 | Randomised trials | Not serious | Serious ^a^ | Not serious | Serious ^c^ | None | 421/3283 (12.8%) | 405/3249 (12.5%) | **OR 1.04**  (0.90 to 1.21) | **4 more per 1,000**  (from 11 fewer to 22 more) | ⨁⨁◯◯ LOW |
| Hospital length of stay | 15 | Randomised trials | Not serious | Serious ^a^ | Not serious | Not serious | None | 4529 | 4535 | - | MD **0.03 days more** (0.3 fewer to 0.3 more) | ⨁⨁⨁◯ MODERATE |
| Surgical site infection | 21 | Randomised trials | Not serious | Serious ^a^ | Not serious | Serious ^d^ | None | 623/7168 (8.7%) | 675/7100 (9.5%) | **OR 0.91**  (0.81 to 1.02) | **8 fewer per 1,000**  (from 17 fewer to 2 more) | ⨁⨁◯◯ LOW |
| Anastomotic leakage | 5 | Randomised trials | Not serious | Not serious | Not serious | Serious ^c^ | None | 49/3687 (1.3%) | 62/3660 (1.7%) | **0.78** (0.54 to 1.14) | **4 fewer per 1,000** (from 8 fewer to 2 more) | ⨁⨁⨁◯ MODERATE |
| Wound dehiscence | 2 | Randomised trials | Not serious | Serious ^a^ | Not serious | Serious ^c^ | None | 49/2915 (1.7%) | 45/2871 (1.6%) | 1.07 (0.71 to 1.62) | **1 fewer per 1,000** (from 4 fewer to 9 more) | ⨁⨁◯◯ LOW |
| Reoperation | 5 | Randomised trials | Not serious | Serious ^a^ | Not serious | Serious ^c^ | None | 130/1083 (12.0%) | 123/1105 (11.1%) | **OR 1.09**  (0.84 to 1.43) | **9 more per 1,000**  (from 16 fewer to 41 more) | ⨁⨁◯◯ LOW |
| Atelectasis | 10 | Randomised trials | Not serious | Serious ^a^ | Not serious | Serious ^b^ | None | 560/5229 (10.7%) | 505/5165 (9.8%) | **OR 1.11**  (0.98 to 1.27) | **10 more per 1,000**  (from 2 fewer to 23 more) | ⨁⨁◯◯ LOW |
| Pneumonia | 9 | Randomised trials | Not serious | Serious ^a^ | Not serious | Serious ^c^ | None | 135/4841 (2.8%) | 146/4786 (3.1%) | **OR 0.92**  (0.72 to 1.17) | **2 fewer per 1,000**  (from 8 fewer to 5 more) | ⨁⨁◯◯ LOW |
| Myocardial infarction/injury | 9 | Randomised trials | Not serious | Serious ^a^ | Not serious | Serious ^c^ | None | 145/3411 (4.3%) | 154/3454 (4.5%) | **OR 0.94**  (0.73 to 1.10) | **3 fewer per 1,000**  (from 12 fewer to 4 more) | ⨁⨁◯◯ LOW |
| *CI: Confidence interval; OR: Odds ratio; MD: Mean difference*  a. Moderate inconsistency (I^2^ = 30-70%)  b. Confidence interval includes both harm and no clinically meaningful effect  c. Confidence interval includes both clinically meaningful harm and benefit  d: Confidence interval includes both benefit and no clinically meaningful effect | | | | | | | | | | | | |

# **FIGURES**

## **Figure S1: PRISMA diagram**

Chart illustrating the flow of studies.

PubMed
(n = 14 836)

EMBASE
(n = 21 951)

Total number of records identified
(n = 36 787)

Records screened
(n = 23 936)

Records excluded (Kappa = 0.61)
(n = 23 393)

Full-text articles assessed for eligibility
(n = 543)

Full-text articles excluded
(n = 513)

No relevant intervention = 235

Not relevant outcome = 188

Other = 28

One lung ventilation = 15

Obstetrics = 11

Not general anaesthesia = 9

Cross over study = 7

Missing full texts = 5

No relevant study type = 5

Paediatric = 3

Very short duration of anaesthesia = 2

Invasive radiology = 1

Data irregularities = 4

Manuscripts included
for the review
(n = 33)

Duplicates excluded
(n = 12 851)

Identified in bibliographies

(n = 3)

## **Figure S2: Long-term mortality, meta-analysis**

**
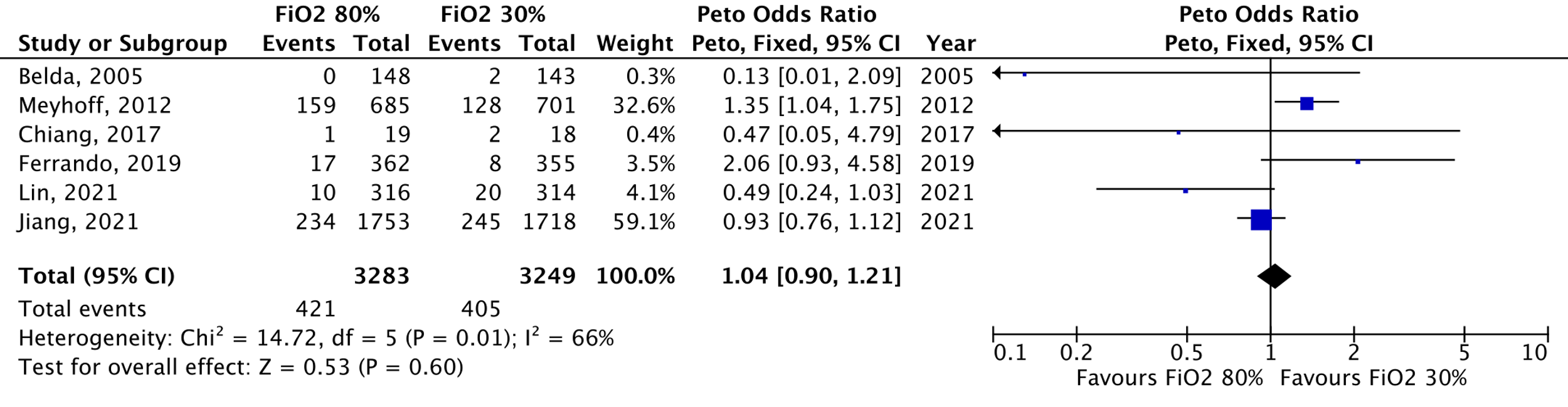
**

*FiO_2_: Fraction of inspired oxygen, CI: confidence interval*

Reported length of long-term mortality ranged from 180-days to 3.2 years

## **Figure S3: Long-term mortality, abdominal surgery subgroup analysis**


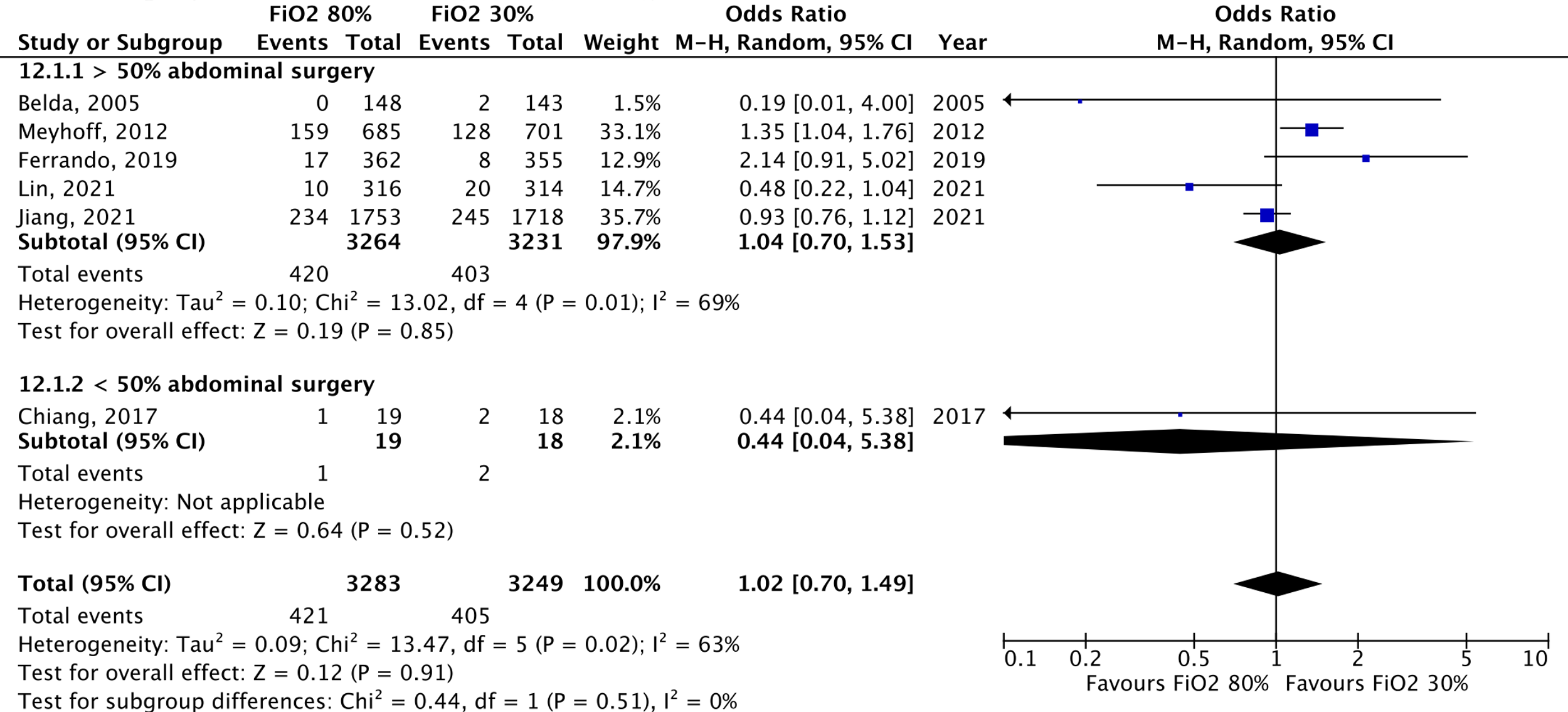


*FiO_2_: Fraction of inspired oxygen, CI: confidence interval*

## **Figure S4: Overall mortality (closets to 30 days), meta-analysis**


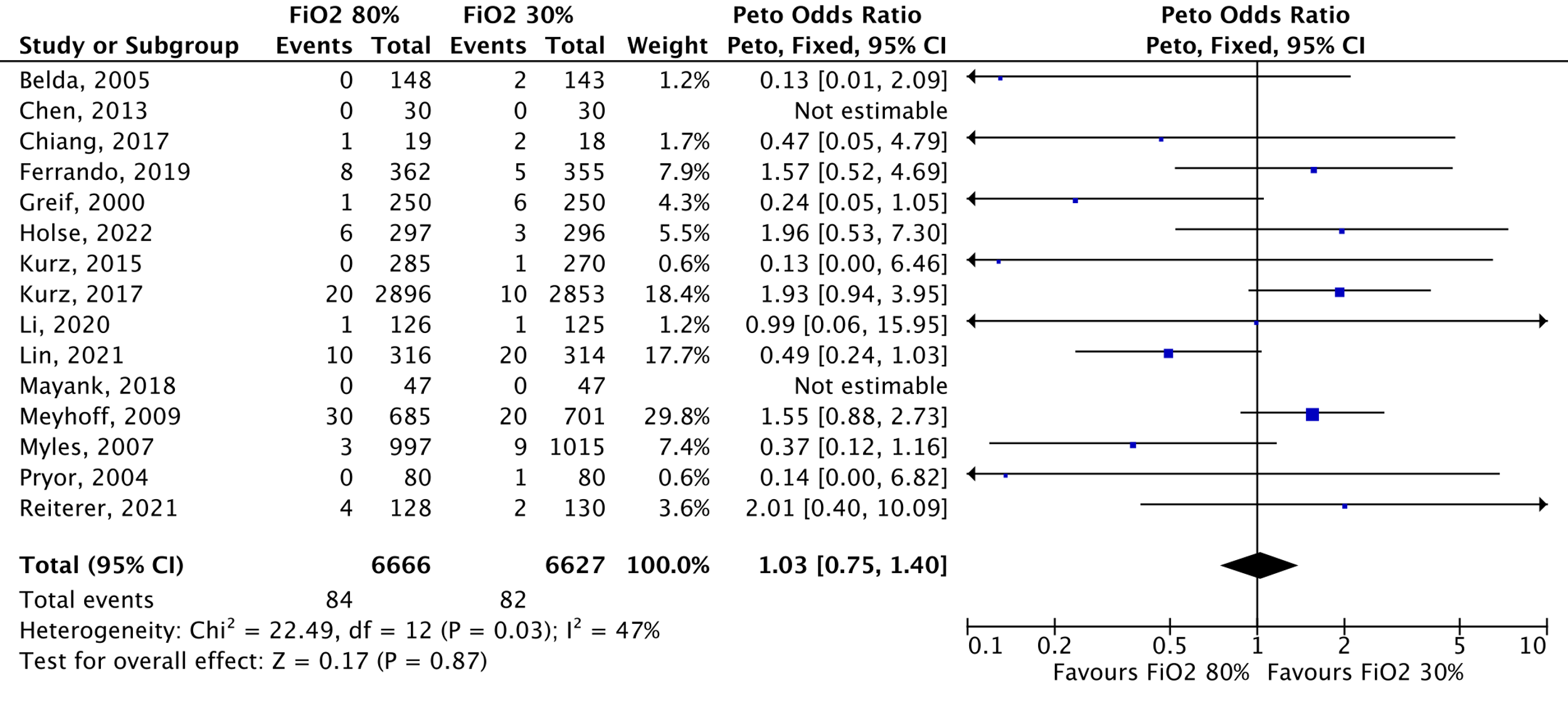


*FiO_2_: Fraction of inspired oxygen, CI: confidence interval*

## **Figure S5: Bubble plot for meta-regression of short-term mortality and median year of patient inclusion**





The Y-axis represents logarithmic odds ratios of the outcome. The X-axis represents the moderator. The circles represent the inverse variance of the study effect sizes. The dotted line represents an odds ratio of 1.0. Values above the dotted line favors FiO_2_ 30% and values below the dotted line favors FiO_2_ 80%. The shaded area represents the 95%

## **Figure S6: Bubble plot for meta-regression of short-term mortality and mortality in the control group**





The Y-axis represents logarithmic odds ratios of the outcome. The X-axis represents the moderator. The circles represent the inverse variance of the study effect sizes. The dotted line represents an odds ratio of 1.0. Values above the dotted line favors FiO_2_ 30% and values below the dotted line favors FiO_2_ 80%. The shaded area represents the 95% confidence intervals.

## **Figure S7: Bubble plot for meta-regression of short-term mortality and sample size**





The Y-axis represents logarithmic odds ratios of the outcome. The X-axis represents the moderator. The circles represent the inverse variance of the study effect sizes. The dotted line represents an odds ratio of 1.0. Values above the dotted line favors FiO_2_ 30% and values below the dotted line favors FiO_2_ 80%. The shaded area represents the 95% confidence intervals.

## **Figure S8: Funnel plot short-term mortality**


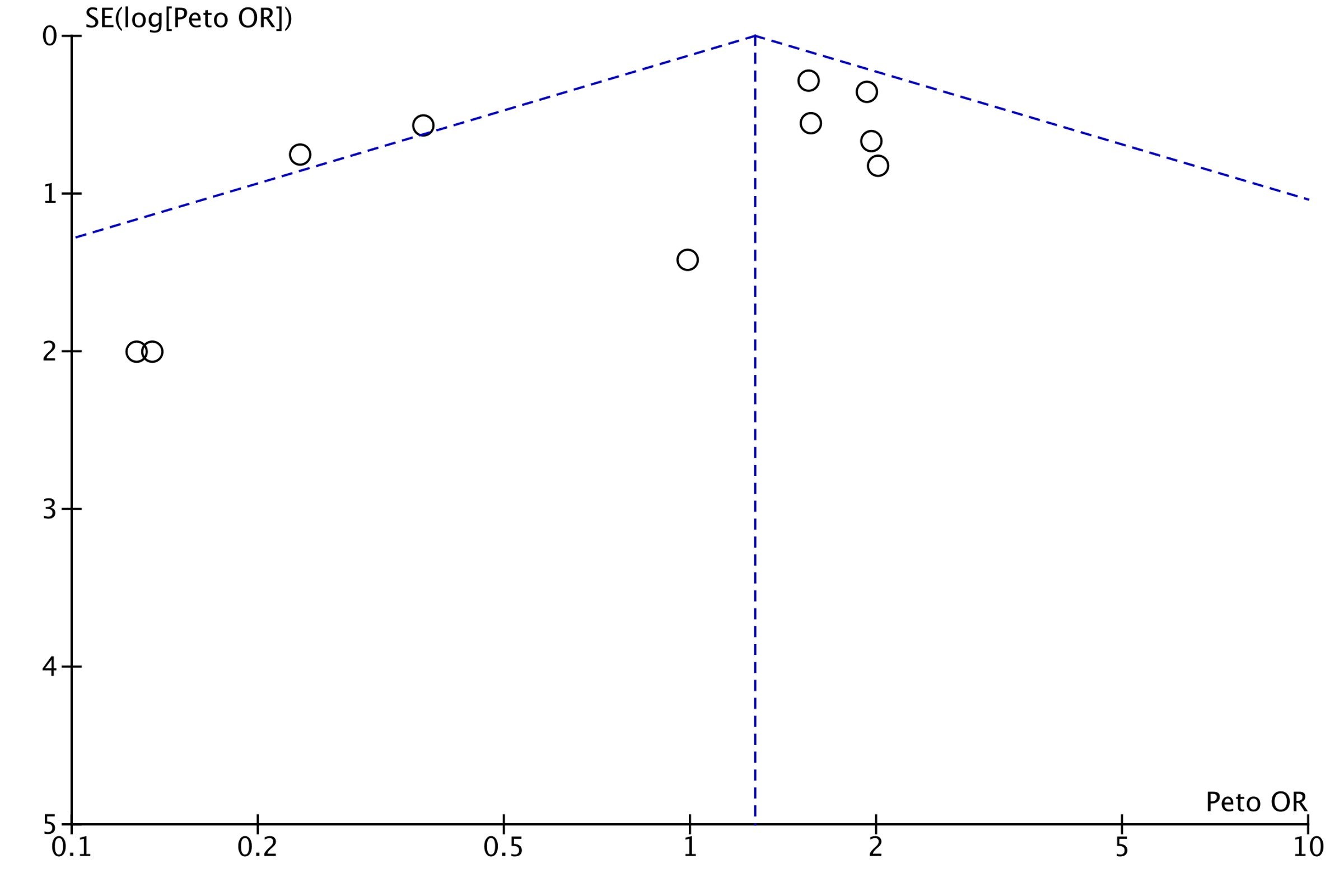


*SE: standard error, OR: odds ratio*

Funnel plot representing risk of publication bias; a plot of odds ratios for short-term mortality in high vs. low FiO_2_ group against its precision (standard error). Every circle represents one clinical trial.

## **Figure S9: Hospital length of stay, meta-analysis**


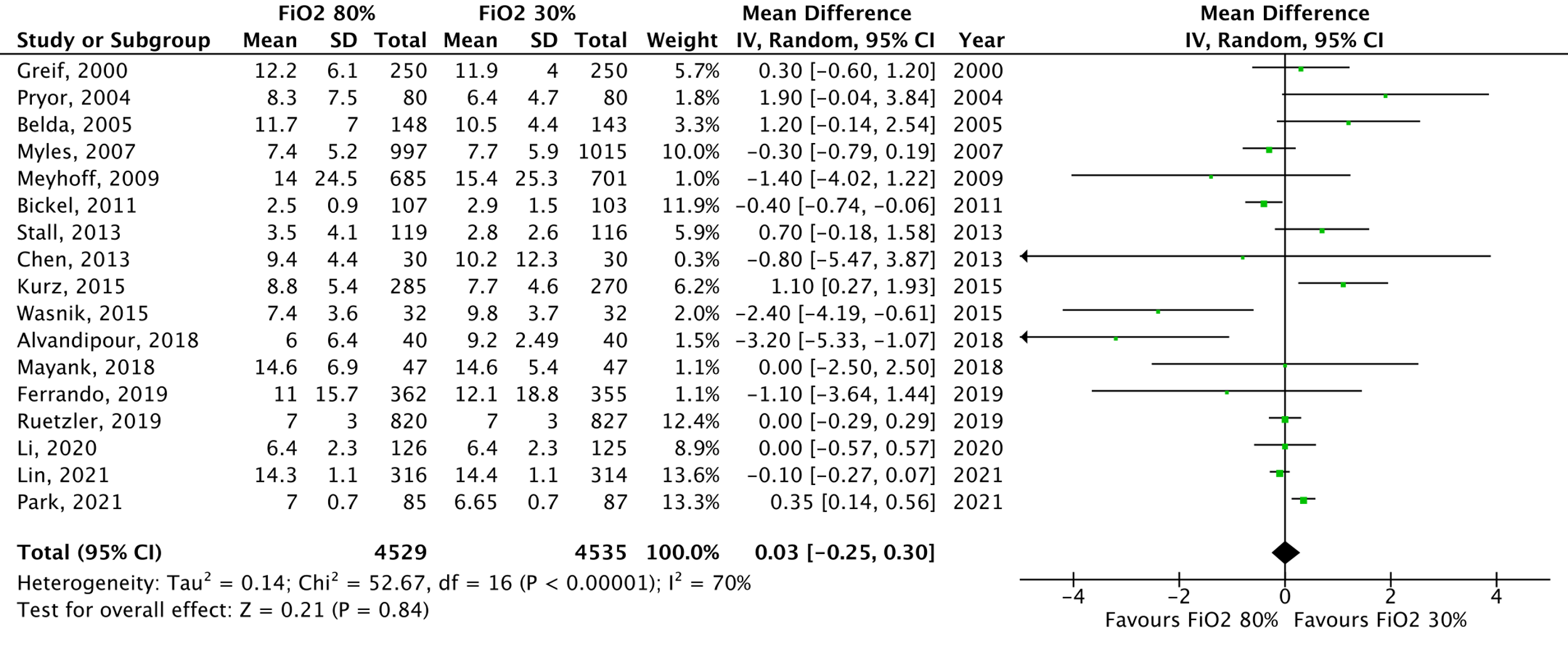


*FiO_2_: Fraction of inspired oxygen, CI: confidence interval*

## **Figure S10: Hospital length of stay, acute surgery subgroup analysis**


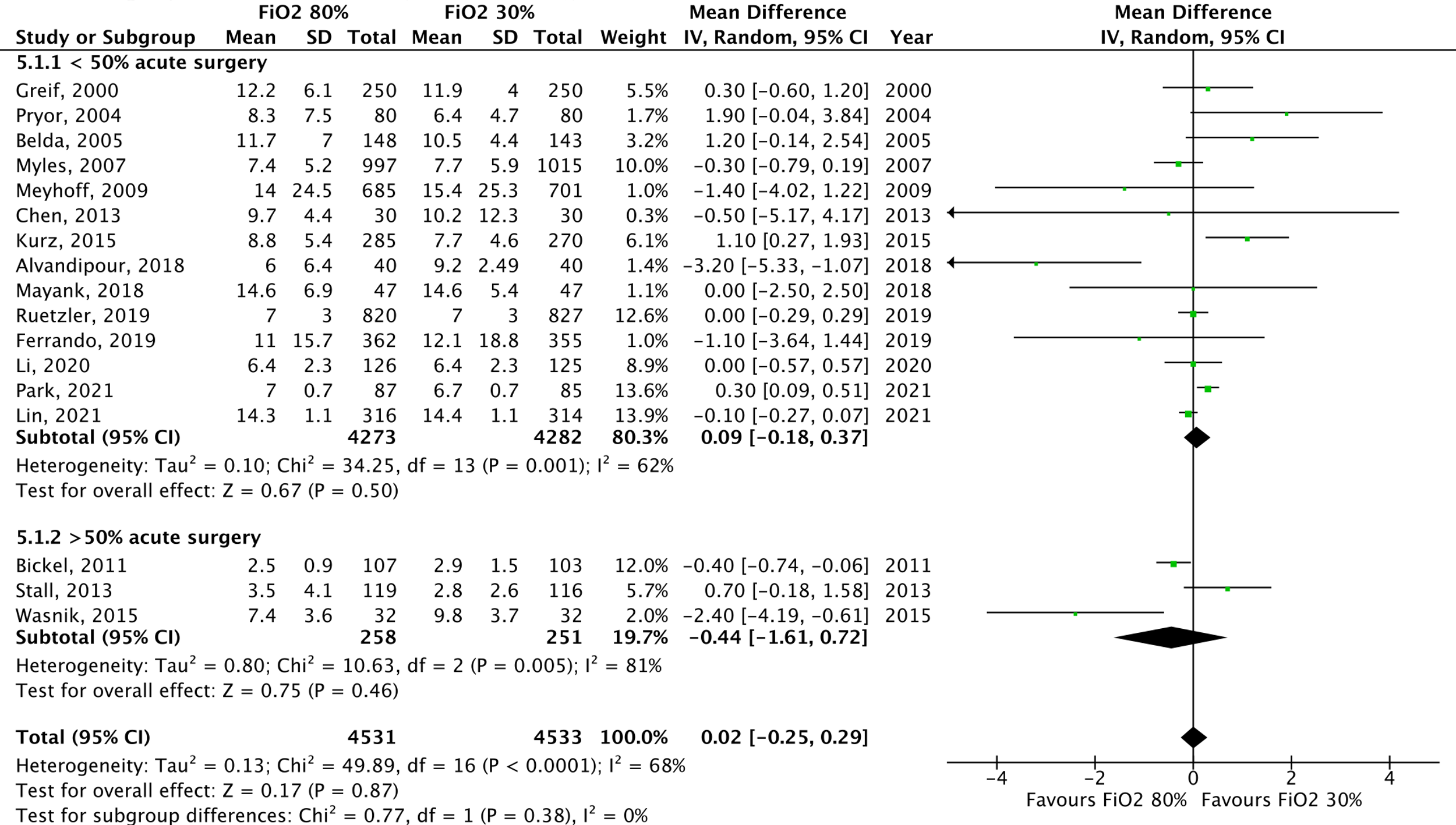


*FiO_2_: Fraction of inspired oxygen, CI: confidence interval*

## **Figure S11: Hospital length of stay, abdominal surgery subgroup analysis**


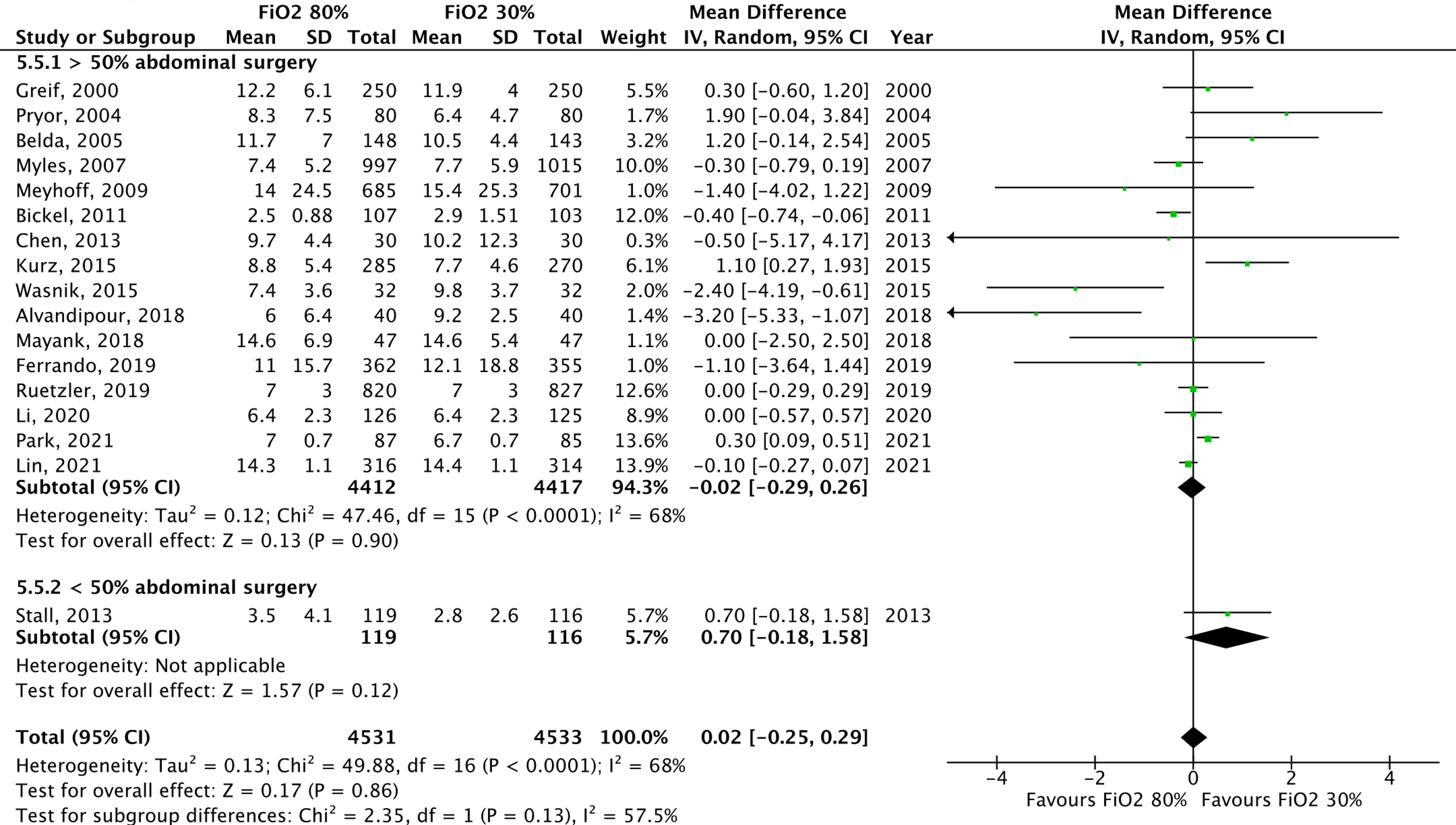


*FiO_2_: Fraction of inspired oxygen, CI: confidence interval*

## **Figure S12: Bubble plot for meta-regression of length of stay and median year of patient inclusion**





The Y-axis represents mean differences of the outcome. The X-axis represents the moderator. The circles represent the inverse variance of the study effect sizes. The dotted line represents an odds ratio of 1.0. Values above the dotted line favors FiO_2_ 30% and values below the dotted line favors FiO_2_ 80%. The shaded area represents the 95% confidence intervals.

## **Figure S13: Bubble plot for meta-regression of length of stay and mortality in the control group**





The Y-axis represents logarithmic odds ratios of the outcome. The X-axis represents the moderator. The circles represent the inverse variance of the study effect sizes. The dotted line represents an odds ratio of 1.0. Values above the dotted line favors FiO_2_ 30% and values below the dotted line favors FiO_2_ 80%. The shaded area represents the 95% confidence intervals.

## **Figure S14: Bubble plot for meta-regression of length of stay and length of stay in the control group**





The Y-axis represents mean differences of the outcome. The X-axis represents the moderator. The circles represent the inverse variance of the study effect sizes. The dotted line represents an odds ratio of 1.0. Values above the dotted line favors FiO_2_ 30% and values below the dotted line favors FiO_2_ 80%. The shaded area represents the 95% confidence intervals.

## **Figure S15: Bubble plot for meta-regression of length of stay and duration of surgery**

**
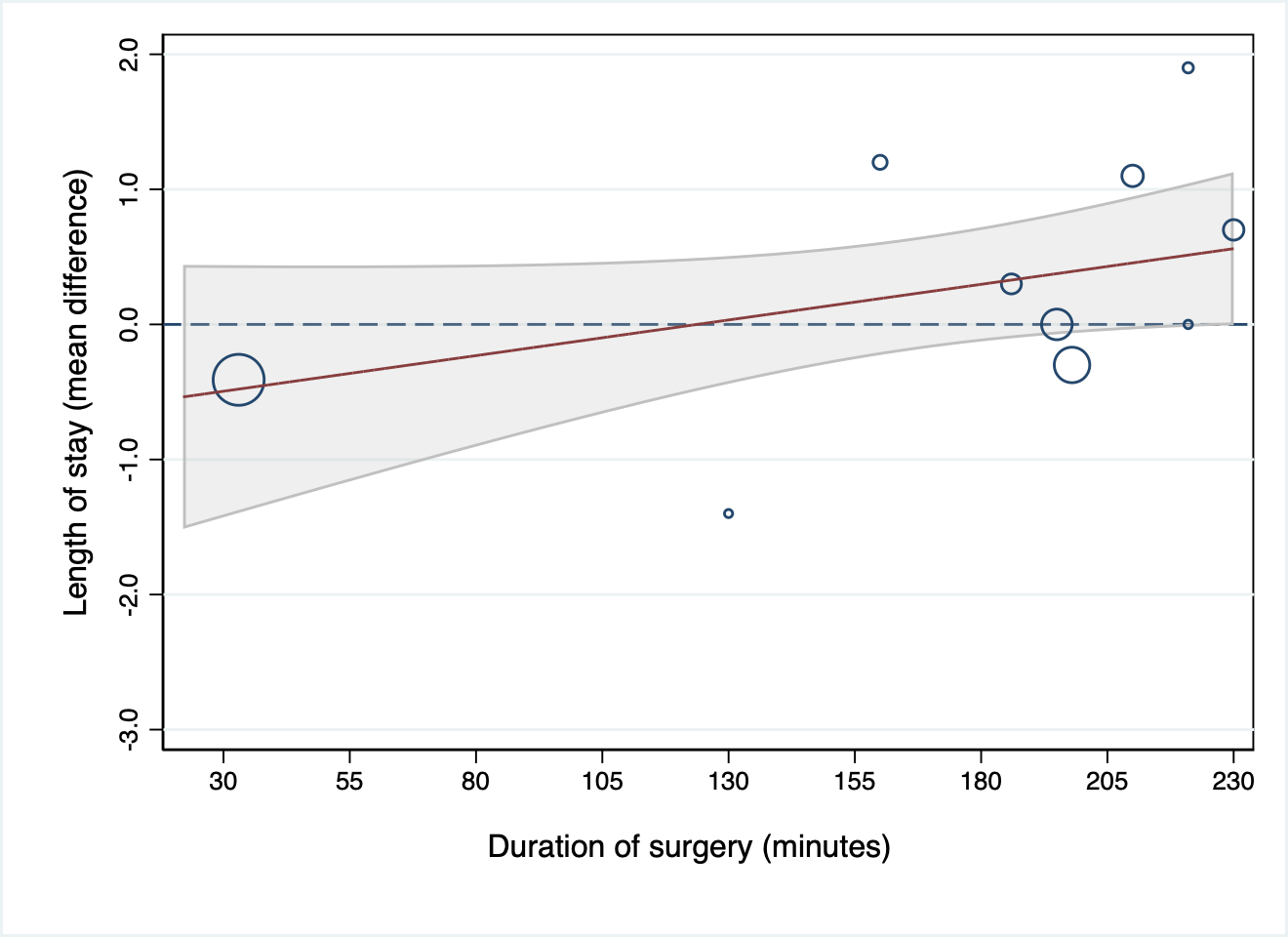
**

The Y-axis represents mean differences of the outcome. The X-axis represents the moderator. The circles represent the inverse variance of the study effect sizes. The dotted line represents an odds ratio of 1.0. Values above the dotted line favors FiO_2_ 30% and values below the dotted line favors FiO_2_ 80%. The shaded area represents the 95% confidence intervals.

## **Figure S16: Bubble plot for meta-regression of length of stay and sample size**





The Y-axis represents mean differences of the outcome. The X-axis represents the moderator. The circles represent the inverse variance of the study effect sizes. The dotted line represents a mean difference of zero. Values above the dotted line favors FiO_2_ 30% and values below the dotted line favors FiO_2_ 80%. The shaded area represents the 95% confidence intervals.

## **Figure S17: Funnel plot hospital length of stay**


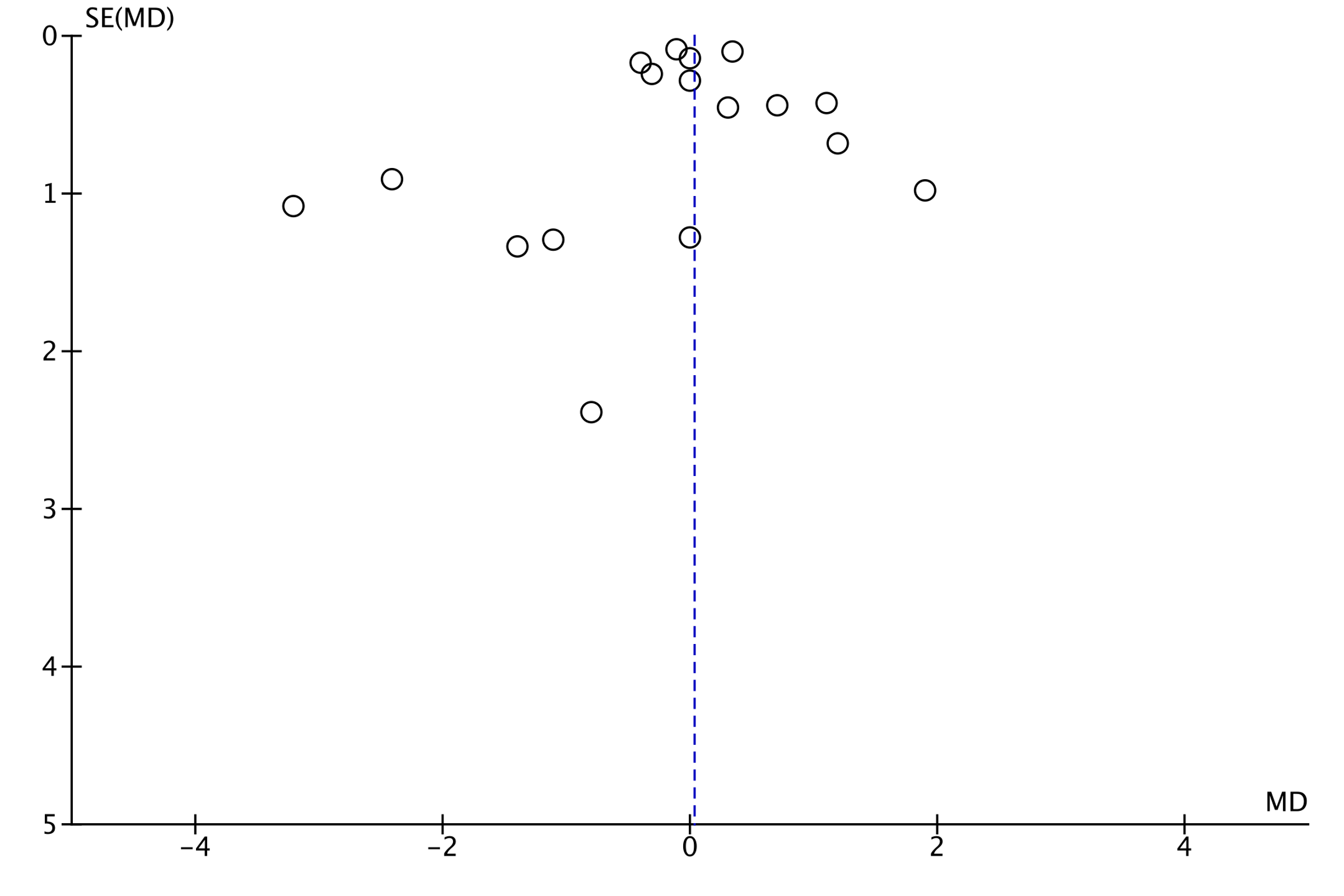


*SE: standard error, OR: odds ratio*

Funnel plot representing risk of publication bias; a plot of odds ratios for hospital length of stay in high vs. low FiO_2_ group against its precision (standard error). Every circle represents one clinical trial.

## **Figure S18: Surgical site infection, acute surgery subgroup analysis**

*
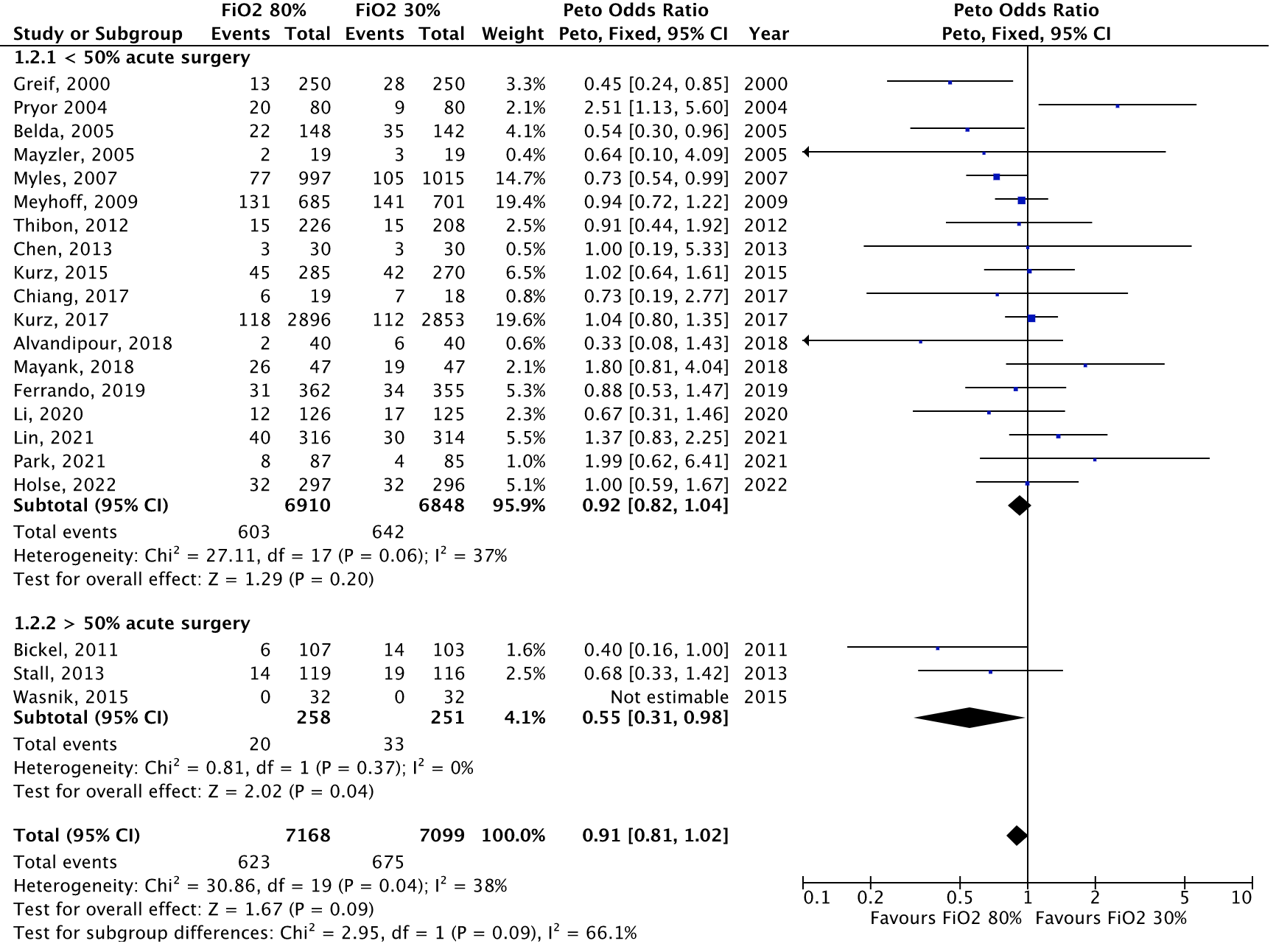
*

*FiO_2_: Fraction of inspired oxygen, CI: confidence interval*

## **Figure S19: Surgical site infection, abdominal surgery subgroup analysis**

**
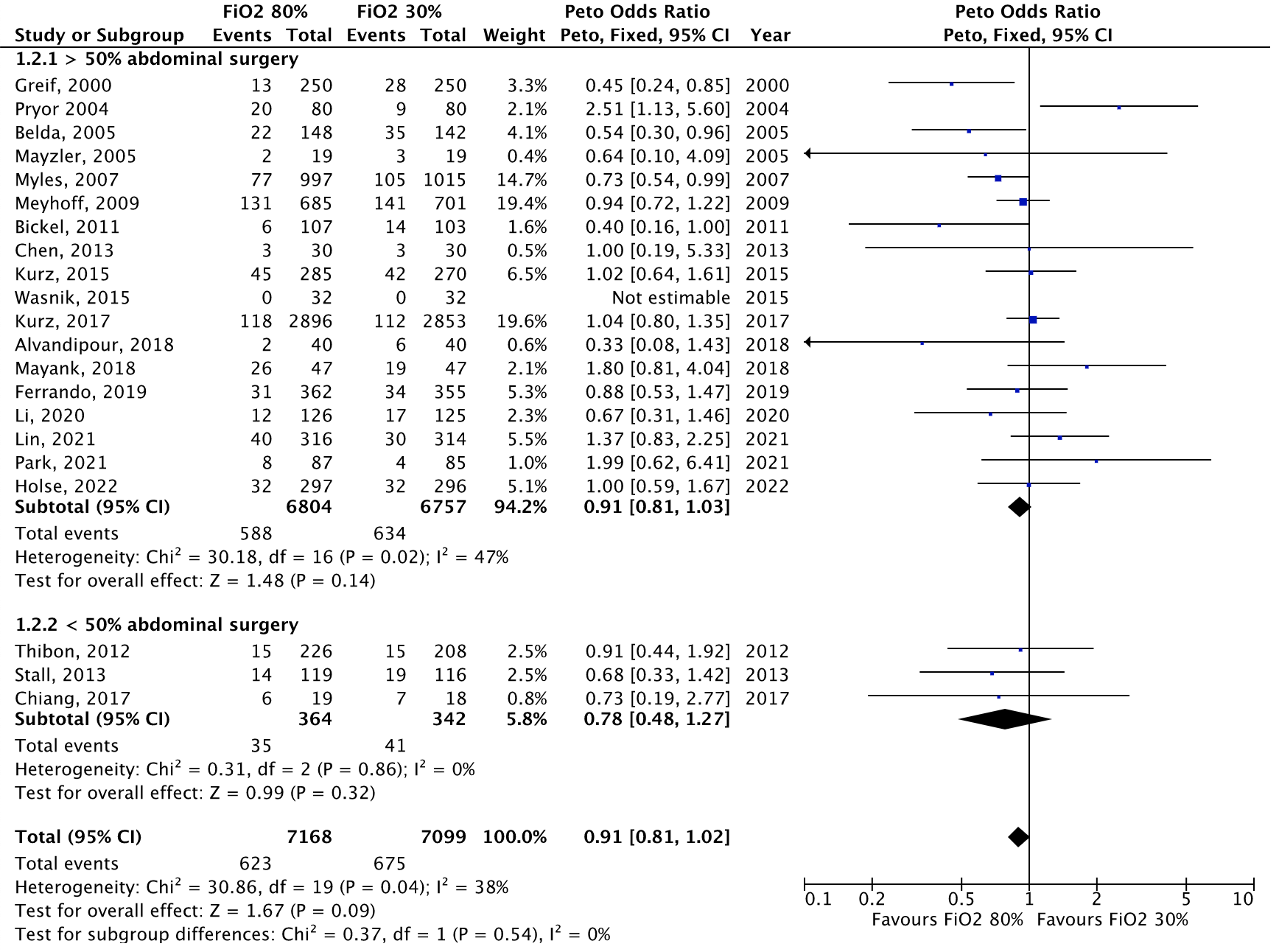
**

*FiO_2_: Fraction of inspired oxygen, CI: confidence interval*

## **Figure S20: Bubble plot for meta-regression of surgical site infection and median year of patient inclusion**

**
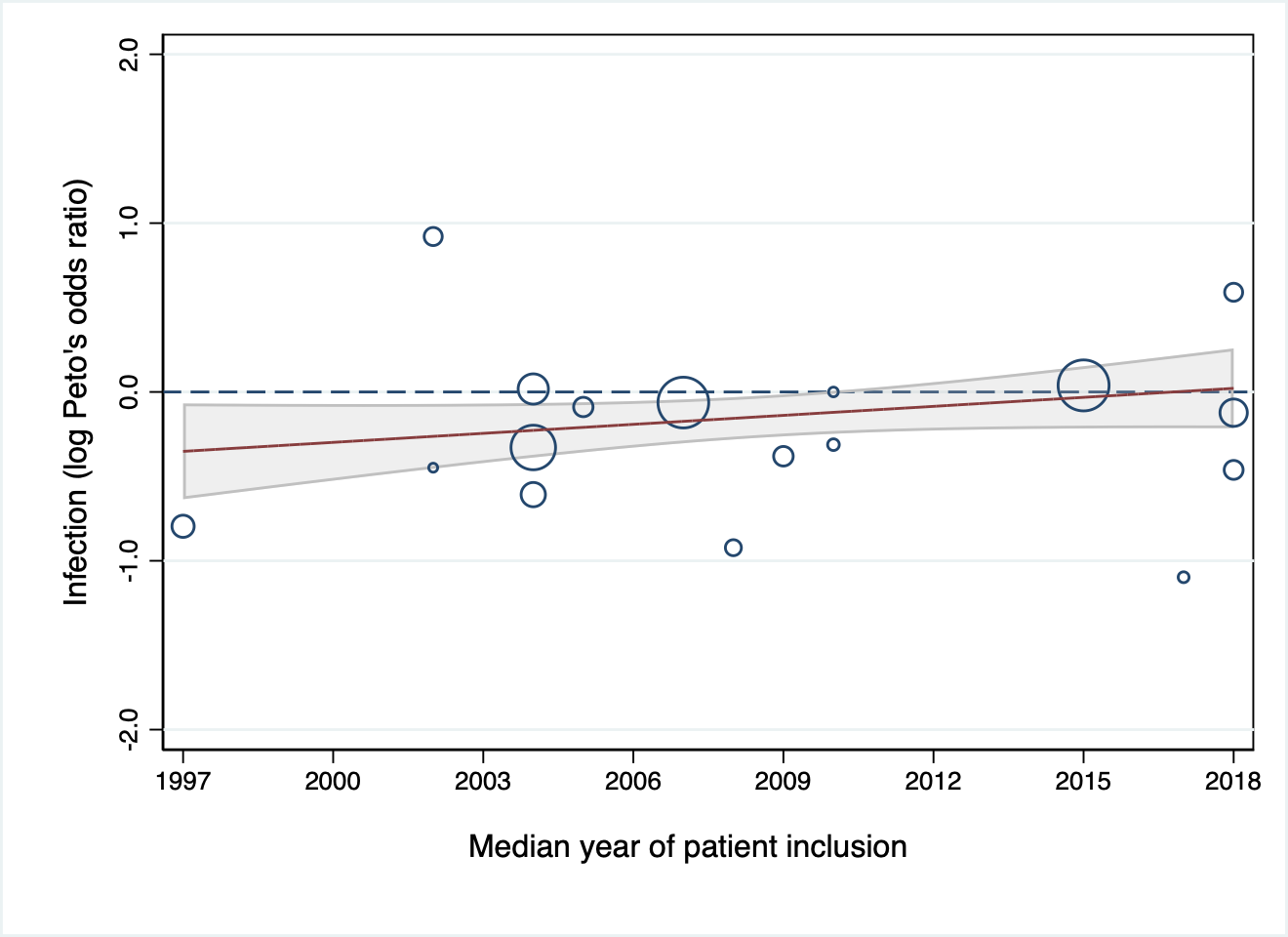
**

The Y-axis represents logarithmic odds ratios of the outcome. The X-axis represents the moderator. The circles represent the inverse variance of the study effect sizes. The dotted line represents an odds ratio of 1.0. Values above the dotted line favors FiO_2_ 30% and values below the dotted line favors FiO_2_ 80%. The shaded area represents the 95% confidence intervals.

## **Figure S21: Bubble plot for meta-regression of surgical site infection and mortality in the control group**

**
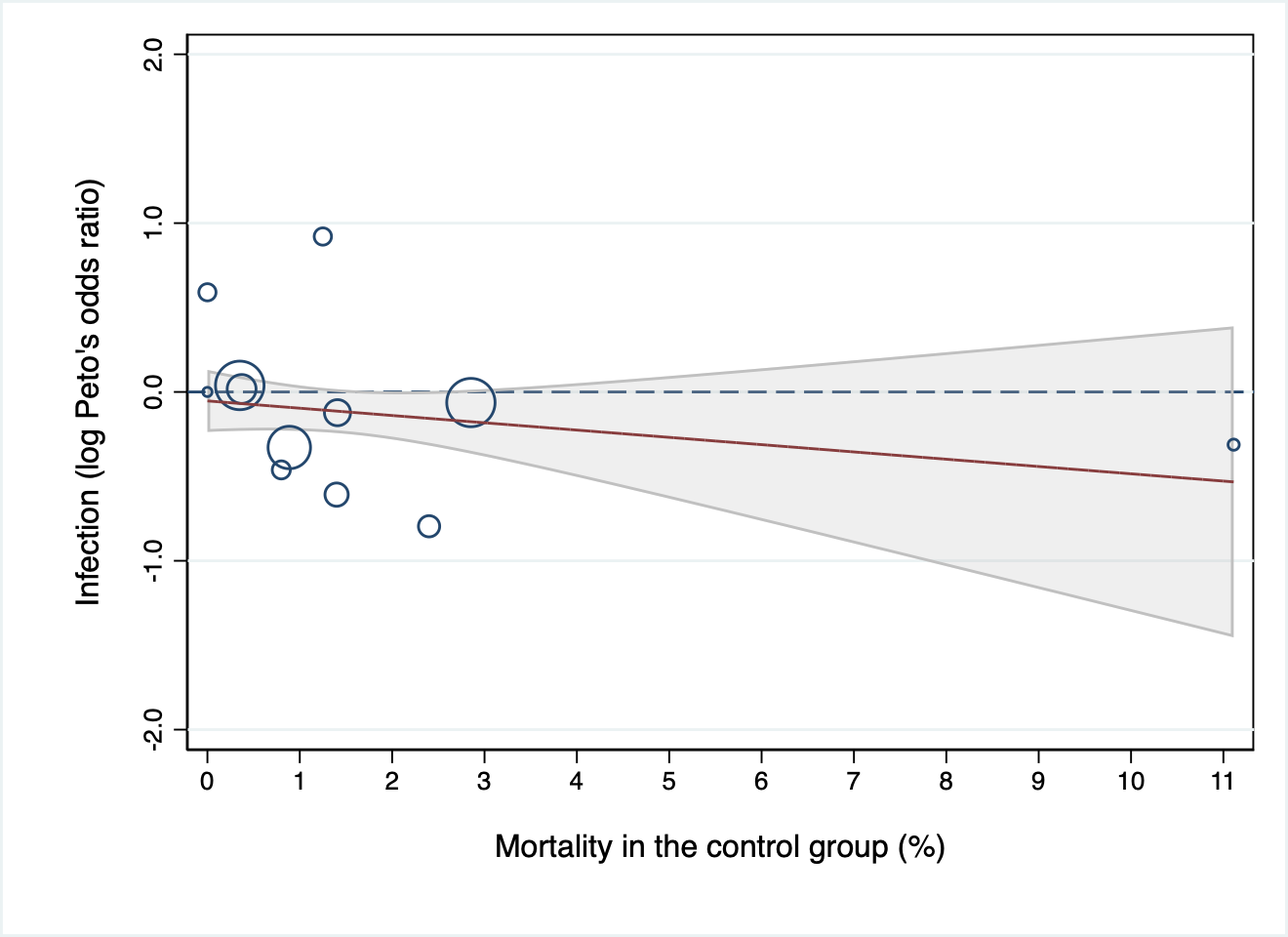
**

The Y-axis represents logarithmic odds ratios of the outcome. The X-axis represents the moderator. The circles represent the inverse variance of the study effect sizes. The dotted line represents an odds ratio of 1.0. Values above the dotted line favors FiO_2_ 30% and values below the dotted line favors FiO_2_ 80%. The shaded area represents the 95% confidence intervals.

## **Figure S22: Bubble plot for meta-regression of surgical site infection and length of stay in the control group**

**
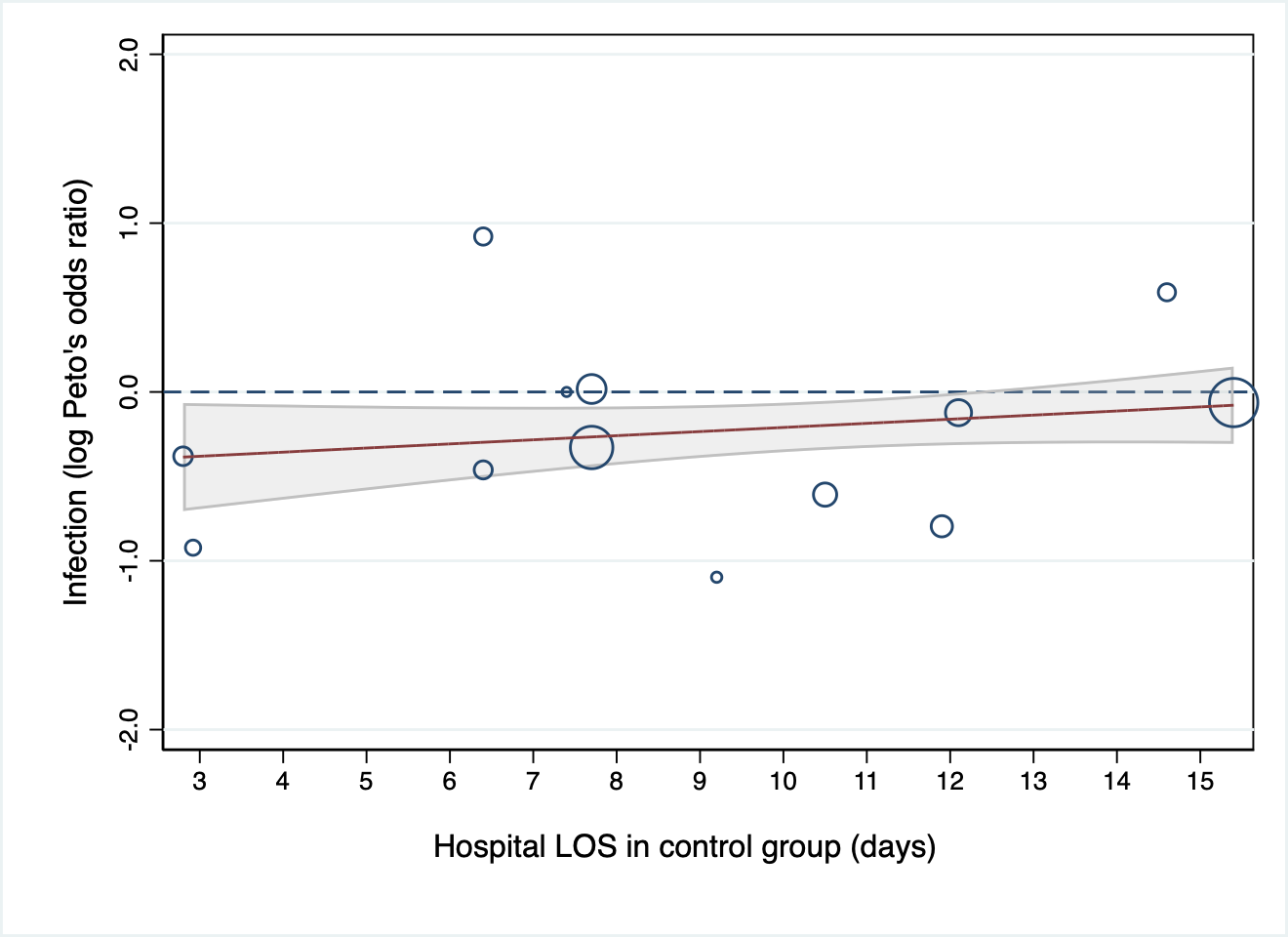
**

The Y-axis represents logarithmic odds ratios of the outcome. The X-axis represents the moderator. The circles represent the inverse variance of the study effect sizes. The dotted line represents an odds ratio of 1.0. Values above the dotted line favors FiO_2_ 30% and values below the dotted line favors FiO_2_ 80%. The shaded area represents the 95% confidence intervals.

**Figure S23: Bubble plot for meta-regression of surgical site infection and duration of surgery**

**
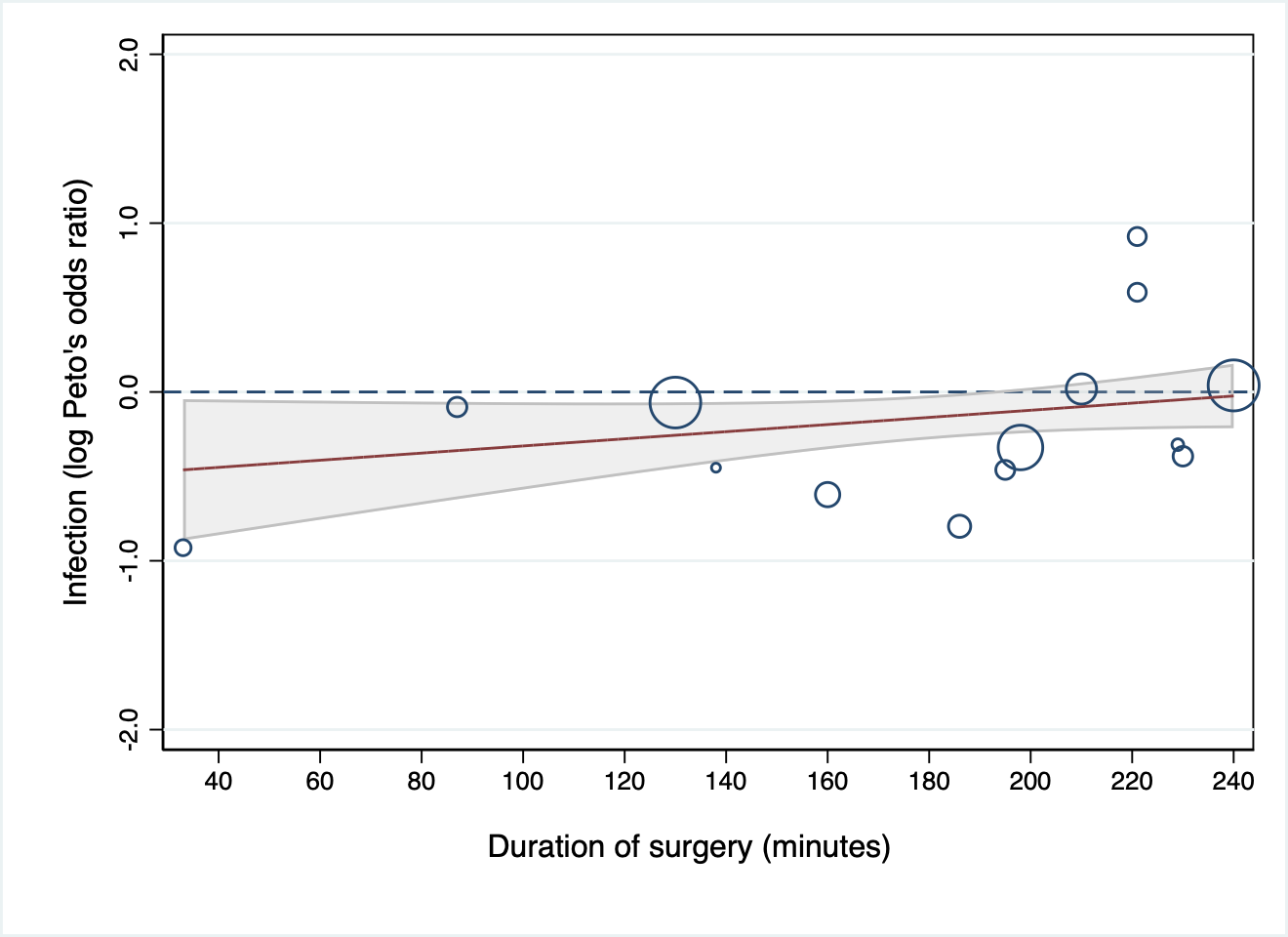
**

The Y-axis represents logarithmic odds ratios of the outcome. The X-axis represents the moderator. The circles represent the inverse variance of the study effect sizes. The dotted line represents an odds ratio of 1.0. Values above the dotted line favors FiO_2_ 30% and values below the dotted line favors FiO_2_ 80%. The shaded area represents the 95% confidence intervals.

## **Figure S24: Bubble plot for meta-regression of surgical site infection and sample size**

**
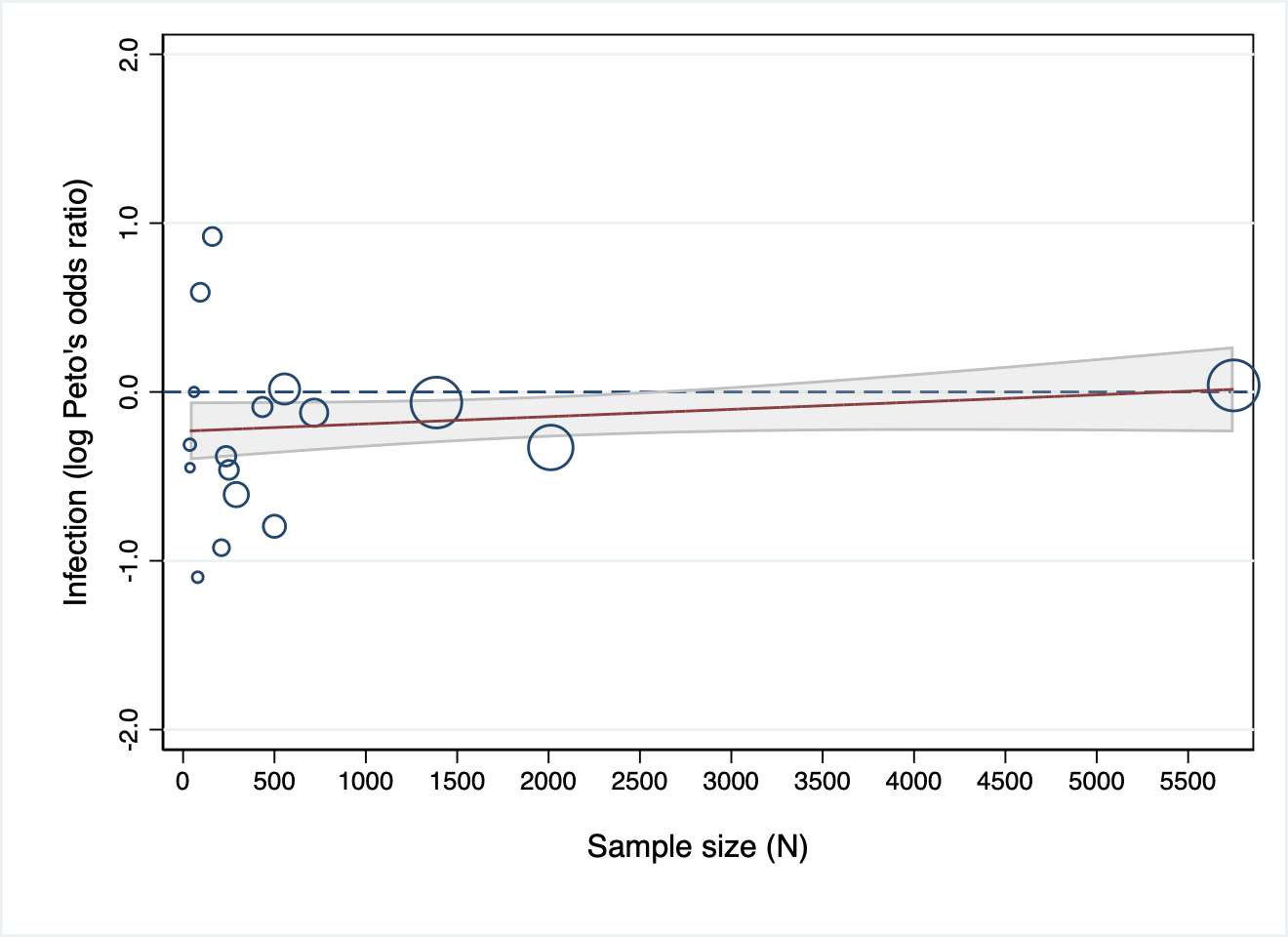
**

The Y-axis represent logarithmic odds ratios of the outcome. The X-axis represent the moderator. The circles represent the inverse variance of the study effect sizes. The dotted line represents an odds ratio of 1.0. Values above the dotted line favors FiO_2_ 80% and values below the dotted line favors FiO_2_ 30%. The Shaded area represents the 95% confidence intervals.

## **Figure S25: Funnel plot surgical site infection**


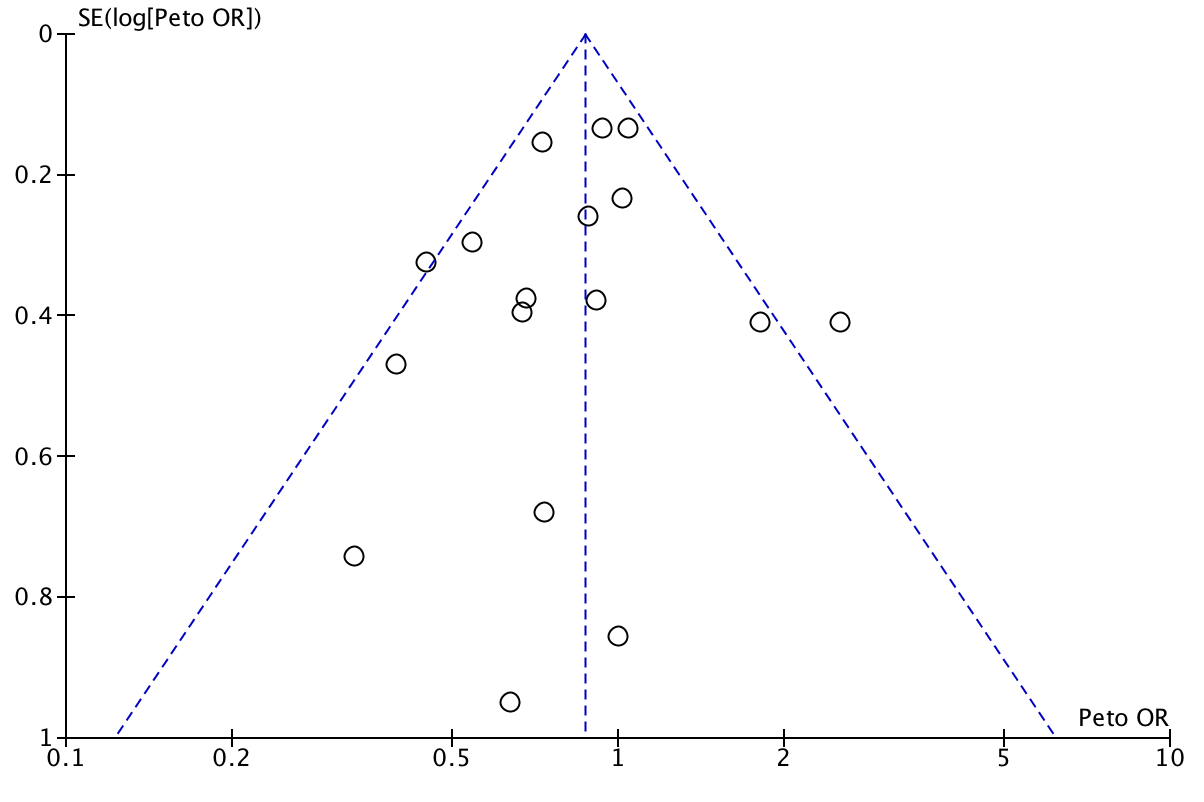


*SE: standard error, OR: odds ratio*

Funnel plot representing risk of publication bias; a plot of odds ratios for surgical site infection in high vs. low FiO_2_ group against its precision (standard error). Every circle represents one clinical trial.

##
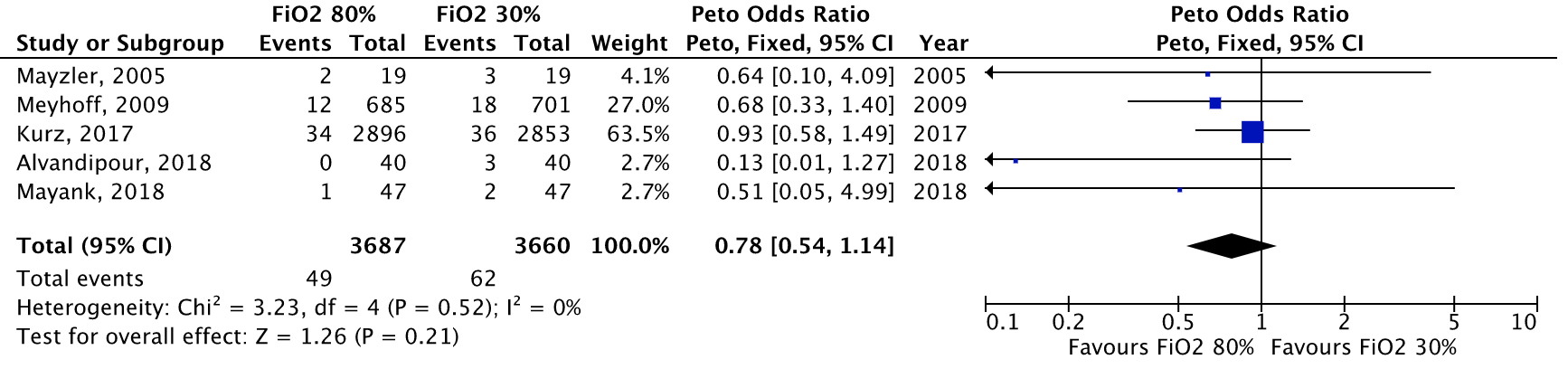
**Figure S26: Anastomotic leakage, meta-analysis**

*FiO_2_: Fraction of inspired oxygen, CI: confidence interval*

## **Figure S27: Wound dehiscence, meta-analysis**


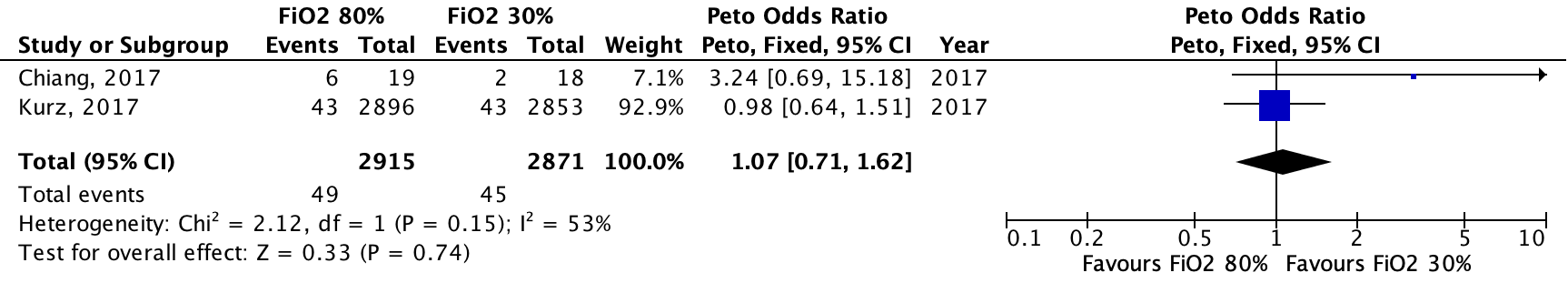


*FiO_2_: Fraction of inspired oxygen, CI: confidence interval*

## **Figure S28: Reoperation, meta-analysis**


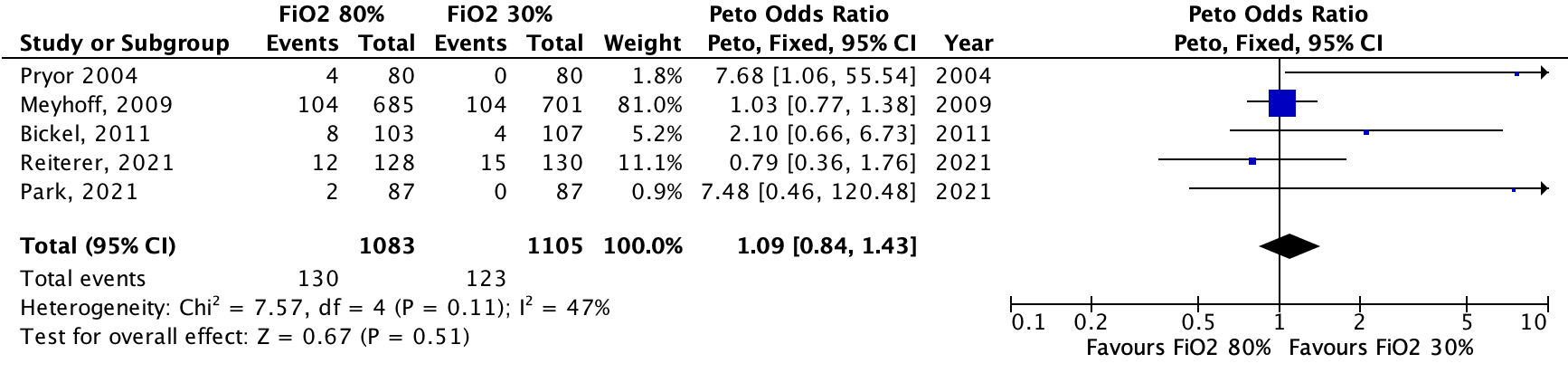


*FiO_2_: Fraction of inspired oxygen, CI: confidence interval*

## **Figure S29: Reoperation, acute surgery subgroup analysis**


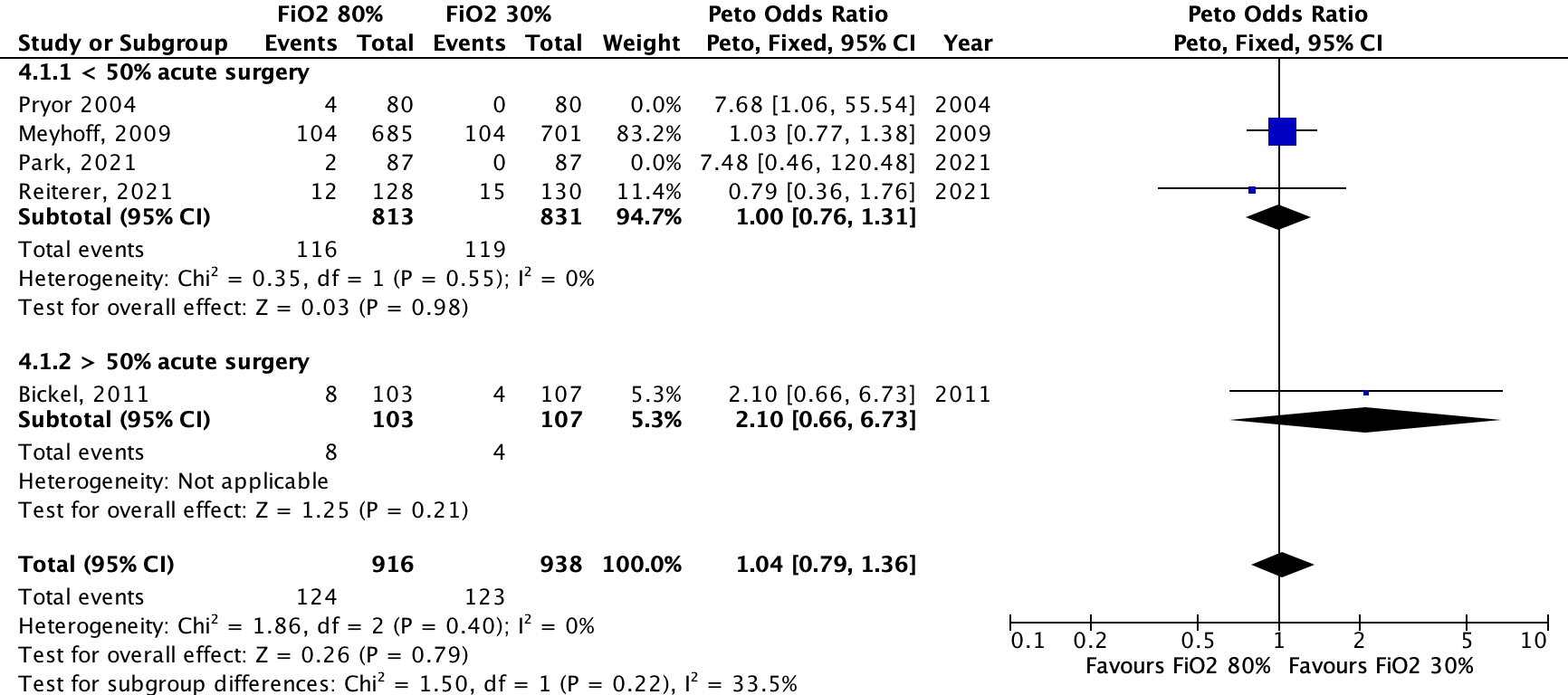


*FiO_2_: Fraction of inspired oxygen, CI: confidence interval*

## **Figure S30: Atelectasis, meta-analysis**


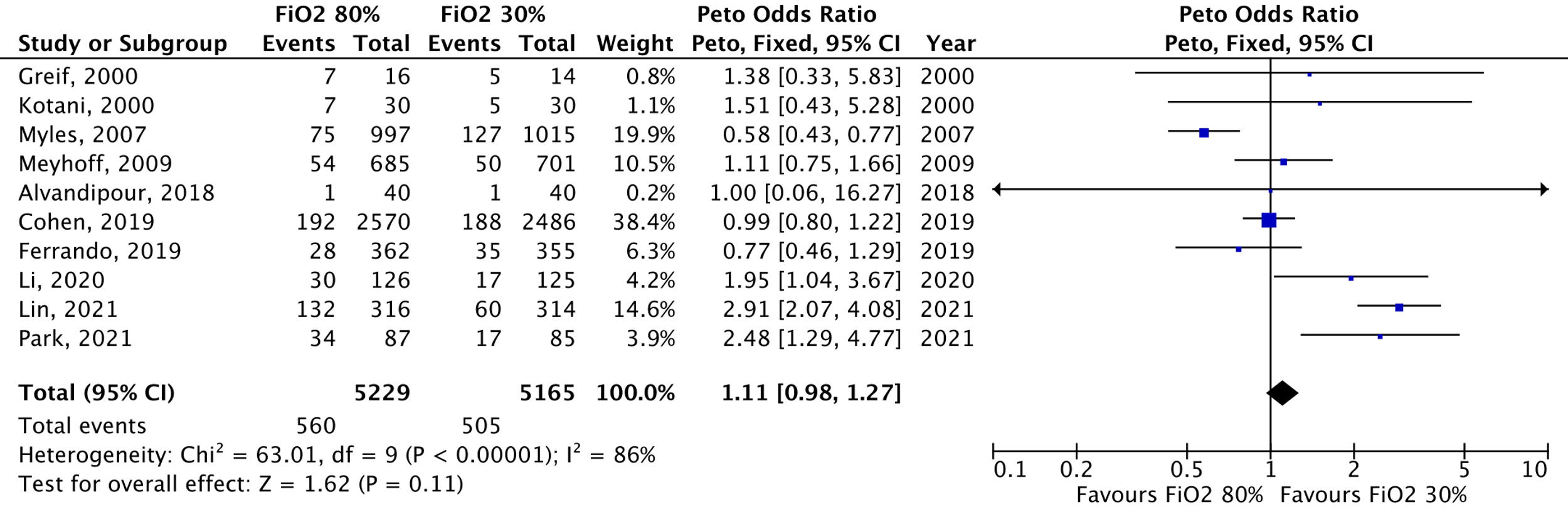


*FiO_2_: Fraction of inspired oxygen, CI: confidence interval*

## **Figure S31: Pneumonia, meta-analysis**


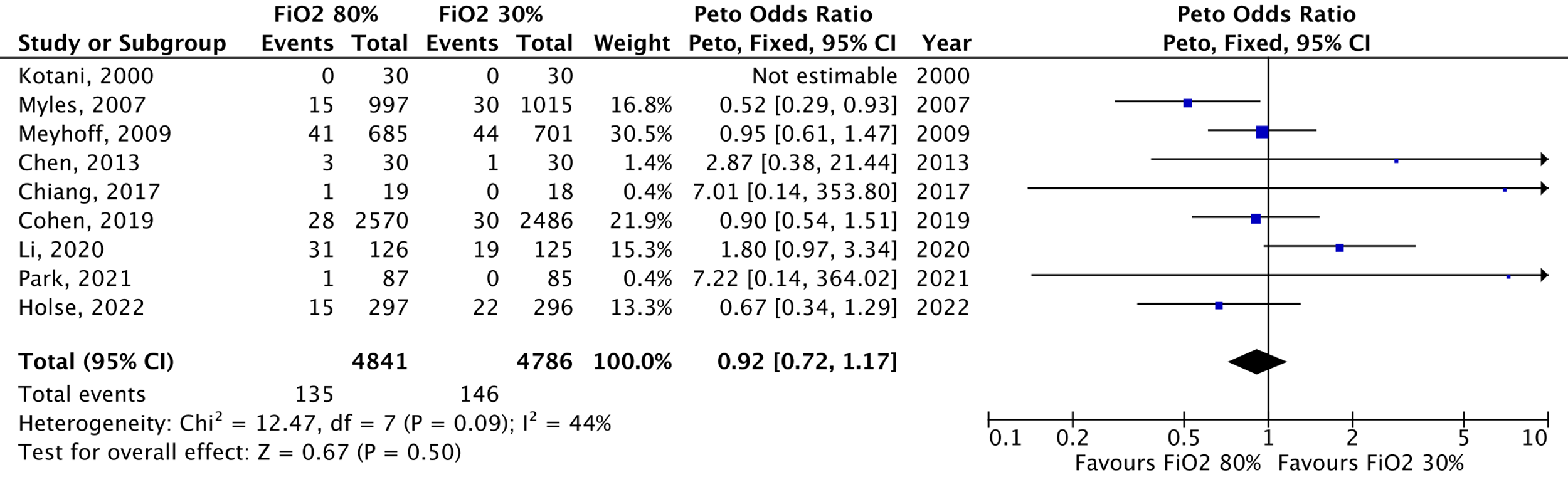


*FiO_2_: Fraction of inspired oxygen, CI: confidence interval*

## **Figure S32: Atelectasis, abdominal surgery subgroup analysis**


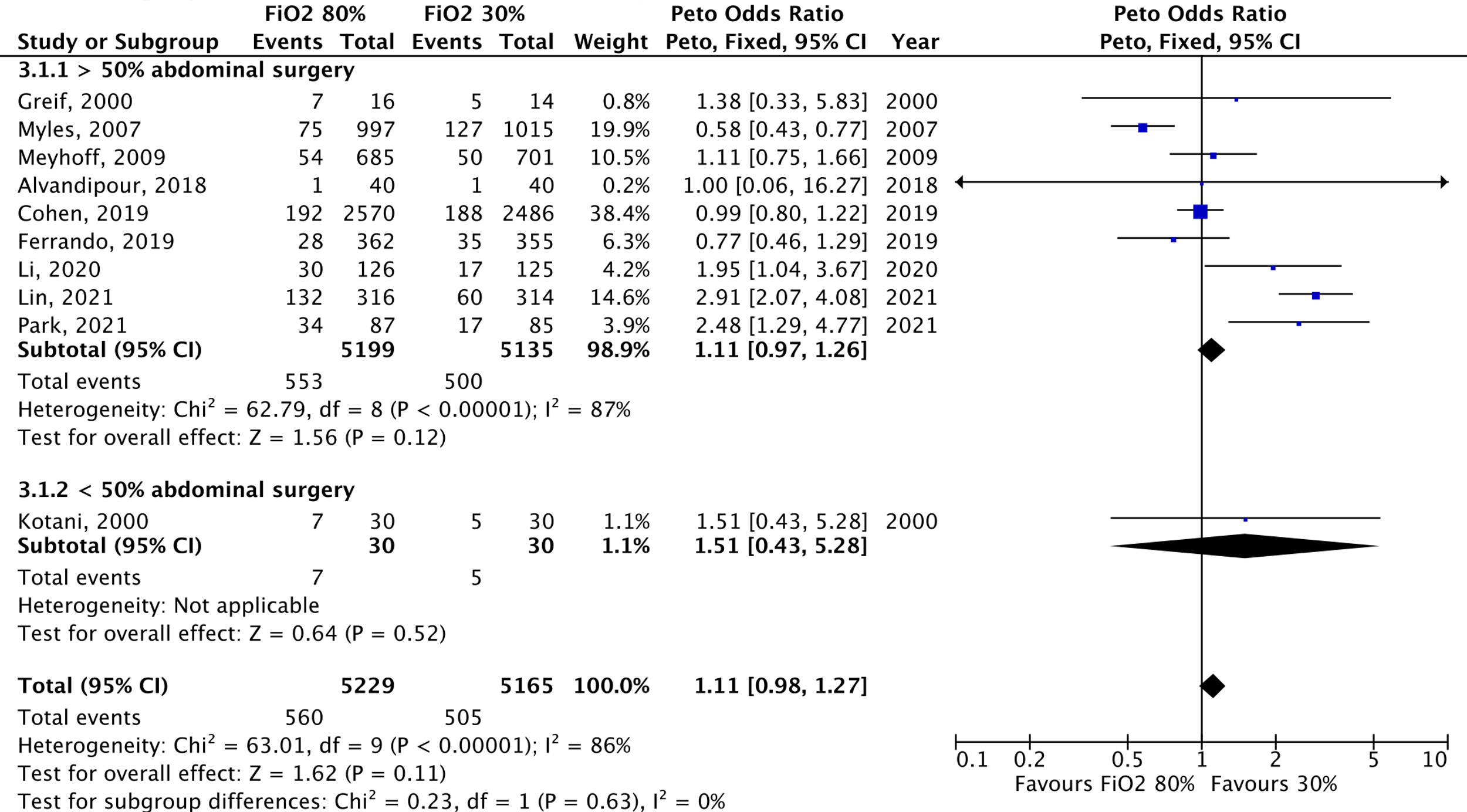


*FiO_2_: Fraction of inspired oxygen, CI: confidence interval*

## **Figure S33: Pneumonia, abdominal surgery subgroup analysis**


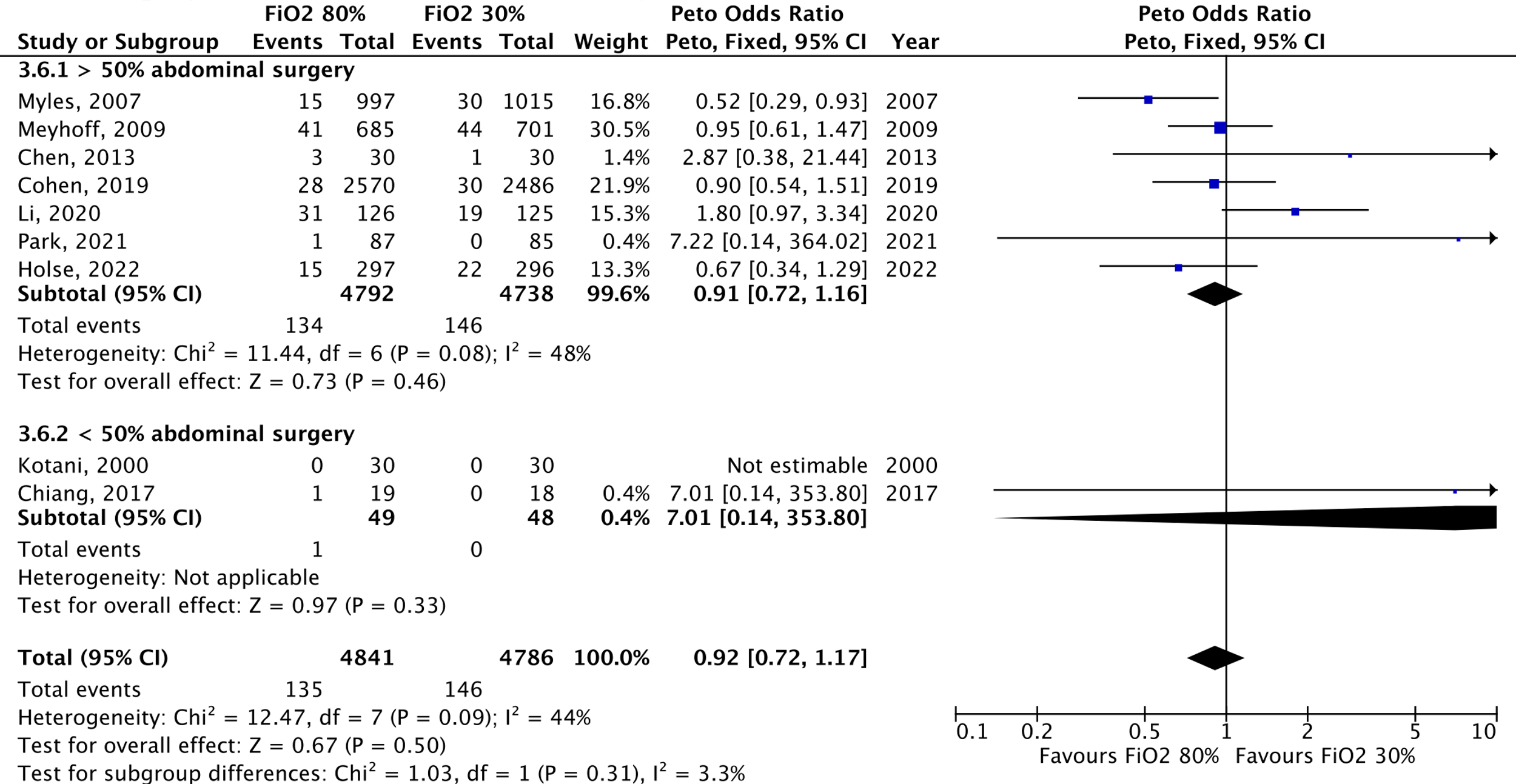


*FiO_2_: Fraction of inspired oxygen, CI: confidence interval*

## **Figure S34: Myocardial injury/myocardial infarction, meta-analysis**


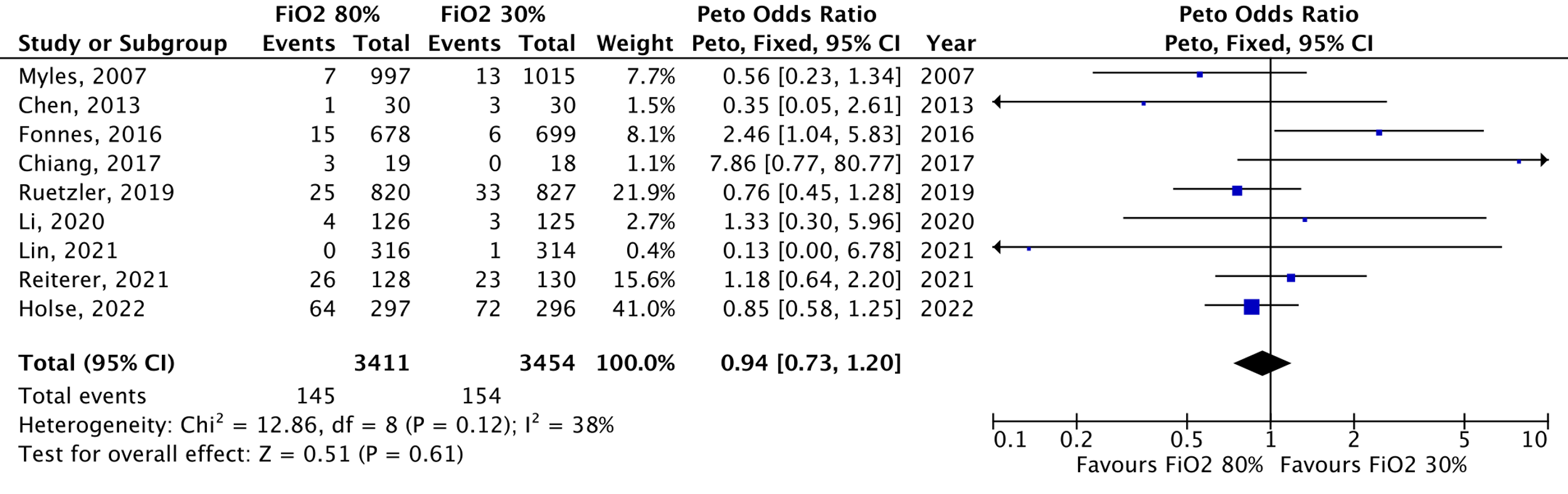


*FiO_2_: Fraction of inspired oxygen, CI: confidence interval*

## **Figure S35: Myocardial injury/myocardial infarction, abdominal surgery subgroup analysis**


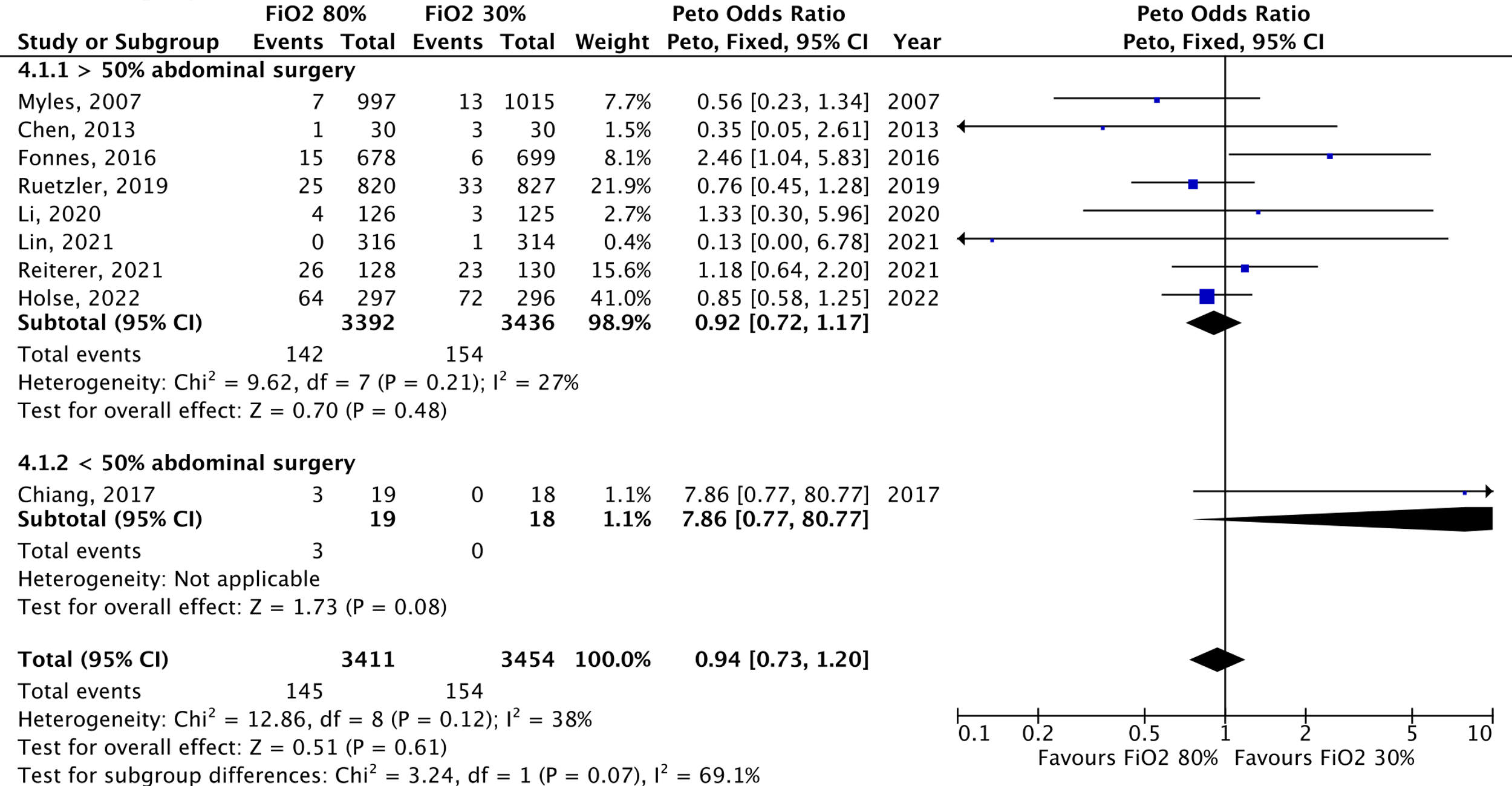


*FiO_2_: Fraction of inspired oxygen, CI: confidence interval*

# **PRISMA CHECKLIST**

| **Section and Topic** | **Item #** | **Checklist item** | **Location where item is reported** |
| --- | --- | --- | --- |
| **TITLE** | | |  |
| Title | 1 | Identify the report as a systematic review. | P1 |
| **ABSTRACT** | | |  |
| Abstract | 2 | See the PRISMA 2020 for Abstracts checklist. | P3 |
| **INTRODUCTION** | | |  |
| Rationale | 3 | Describe the rationale for the review in the context of existing knowledge. | P4 |
| Objectives | 4 | Provide an explicit statement of the objective(s) or question(s) the review addresses. | P4 |
| **METHODS** | | |  |
| Eligibility criteria | 5 | Specify the inclusion and exclusion criteria for the review and how studies were grouped for the syntheses. | P5 |
| Information sources | 6 | Specify all databases, registers, websites, organizations, reference lists and other sources searched or consulted to identify studies. Specify the date when each source was last searched or consulted. | P5 |
| Search strategy | 7 | Present the full search strategies for all databases, registers and websites, including any filters and limits used. | Protocol |
| Selection process | 8 | Specify the methods used to decide whether a study met the inclusion criteria of the review, including how many reviewers screened each record and each report retrieved, whether they worked independently, and if applicable, details of automation tools used in the process. | P6 |
| Data collection process | 9 | Specify the methods used to collect data from reports, including how many reviewers collected data from each report, whether they worked independently, any processes for obtaining or confirming data from study investigators, and if applicable, details of automation tools used in the process. | P6 |
| Data items | 10a | List and define all outcomes for which data were sought. Specify whether all results that were compatible with each outcome domain in each study were sought (e.g. for all measures, time points, analyses), and if not, the methods used to decide which results to collect. | P4-5  Supplement |
|  | 10b | List and define all other variables for which data were sought (e.g. participant and intervention characteristics, funding sources). Describe any assumptions made about any missing or unclear information. | Supplement |
| Study risk of bias assessment | 11 | Specify the methods used to assess risk of bias in the included studies, including details of the tool(s) used, how many reviewers assessed each study and whether they worked independently, and if applicable, details of automation tools used in the process. | P6  Supplement |
| Effect measures | 12 | Specify for each outcome the effect measure(s) (e.g. risk ratio, mean difference) used in the synthesis or presentation of results. | P6-7 |
| Synthesis methods | 13a | Describe the processes used to decide which studies were eligible for each synthesis (e.g. tabulating the study intervention characteristics and comparing against the planned groups for each synthesis (item #5)). | P7 |
|  | 13b | Describe any methods required to prepare the data for presentation or synthesis, such as handling of missing summary statistics, or data conversions. | P6-7 |
|  | 13c | Describe any methods used to tabulate or visually display results of individual studies and syntheses. | Tables and figures |
|  | 13d | Describe any methods used to synthesize results and provide a rationale for the choice(s). If meta-analysis was performed, describe the model(s), method(s) to identify the presence and extent of statistical heterogeneity, and software package(s) used. | P6-7 |
|  | 13e | Describe any methods used to explore possible causes of heterogeneity among study results (e.g. subgroup analysis, meta-regression). | P7 |
|  | 13f | Describe any sensitivity analyses conducted to assess robustness of the synthesized results. | P7 |
| Reporting bias assessment | 14 | Describe any methods used to assess risk of bias due to missing results in a synthesis (arising from reporting biases). | P7 |
| Certainty assessment | 15 | Describe any methods used to assess certainty (or confidence) in the body of evidence for an outcome. | P7 |
| **RESULTS** | | |  |
| Study selection | 16a | Describe the results of the search and selection process, from the number of records identified in the search to the number of studies included in the review, ideally using a flow diagram. | P8 |
|  | 16b | Cite studies that might appear to meet the inclusion criteria, but which were excluded, and explain why they were excluded. | Figure S1 |
| Study characteristics | 17 | Cite each included study and present its characteristics. | Table 1 |
| Risk of bias in studies | 18 | Present assessments of risk of bias for each included study. | Table S4 |
| Results of individual studies | 19 | For all outcomes, present, for each study: (a) summary statistics for each group (where appropriate) and (b) an effect estimate and its precision (e.g. confidence/credible interval), ideally using structured tables or plots. | Figures |
| Results of syntheses | 20a | For each synthesis, briefly summarize the characteristics and risk of bias among contributing studies. | Table 1, Table S2, Table S4 |
|  | 20b | Present results of all statistical syntheses conducted. If meta-analysis was done, present for each the summary estimate and its precision (e.g. confidence/credible interval) and measures of statistical heterogeneity. If comparing groups, describe the direction of the effect. | Figure 1 |
|  | 20c | Present results of all investigations of possible causes of heterogeneity among study results. | Tables and figures |
|  | 20d | Present results of all sensitivity analyses conducted to assess the robustness of the synthesized results. | Table S6 |
| Reporting biases | 21 | Present assessments of risk of bias due to missing results (arising from reporting biases) for each synthesis assessed. |  |
| Certainty of evidence | 22 | Present assessments of certainty (or confidence) in the body of evidence for each outcome assessed. | Table S7 |
| **DISCUSSION** | | |  |
| Discussion | 23a | Provide a general interpretation of the results in the context of other evidence. | P11 |
|  | 23b | Discuss any limitations of the evidence included in the review. | P13 |
|  | 23c | Discuss any limitations of the review processes used. | P11 |
|  | 23d | Discuss implications of the results for practice, policy, and future research. | P13 |
| **OTHER INFORMATION** | | |  |
| Registration and protocol | 24a | Provide registration information for the review, including register name and registration number, or state that the review was not registered. | P4 |
|  | 24b | Indicate where the review protocol can be accessed, or state that a protocol was not prepared. | P4 |
|  | 24c | Describe and explain any amendments to information provided at registration or in the protocol. | P5 |
| Support | 25 | Describe sources of financial or non-financial support for the review, and the role of the funders or sponsors in the review. | P14 |
| Competing interests | 26 | Declare any competing interests of review authors. | P14 |
| Availability of data, code and other materials | 27 | Report which of the following are publicly available and where they can be found: template data collection forms; data extracted from included studies; data used for all analyses; analytic code; any other materials used in the review. | NA |

# **REFERENCES**

1 Horan, T. C., Gaynes, R. P., Martone, W. J., Jarvis, W. R. & Emori, T. G. CDC definitions of nosocomial surgical site infections, 1992: a modification of CDC definitions of surgical wound infections. *Infect Control Hosp Epidemiol* **13**, 606-608 (1992).

2 Wilson, A. P., Treasure, T., Sturridge, M. F. & Grüneberg, R. N. A scoring method (ASEPSIS) for postoperative wound infections for use in clinical trials of antibiotic prophylaxis. *Lancet* **1**, 311-313, doi:10.1016/s0140-6736(86)90838-x (1986).

3 Prevention, C. f. D. C. a. *Guidelines for Prevention of Nosocomial Pneumonia*, <<https://www.cdc.gov/mmwr/preview/mmwrhtml/00045365.htm>> (1997).

4 Canet, J. *et al.* Prediction of postoperative pulmonary complications in a population-based surgical cohort. *Anesthesiology* **113**, 1338-1350, doi:10.1097/ALN.0b013e3181fc6e0a (2010).

5 Julian Higgins, J. T. Revised Cochrane risk-of-bias tool for randomized trials (RoB 2) (2019).

6 Guyatt, G. H. *et al.* GRADE guidelines 6. Rating the quality of evidence--imprecision. *J Clin Epidemiol* **64**, 1283-1293, doi:10.1016/j.jclinepi.2011.01.012 (2011).

7 Kotani, N. *et al.* Supplemental intraoperative oxygen augments antimicrobial and proinflammatory responses of alveolar macrophages. *Anesthesiology* **93**, 15-25, doi:10.1097/00000542-200007000-00008 (2000).

8 Greif, R., Akça, O., Horn, E. P., Kurz, A. & Sessler, D. I. Supplemental perioperative oxygen to reduce the incidence of surgical-wound infection. *N Engl J Med* **342**, 161-167, doi:10.1056/nejm200001203420303 (2000).

9 Purhonen, S., Turunen, M., Ruohoaho, U. M., Niskanen, M. & Hynynen, M. Supplemental oxygen does not reduce the incidence of postoperative nausea and vomiting after ambulatory gynecologic laparoscopy. *Anesth Analg* **96**, 91-96, table of contents, doi:10.1097/00000539-200301000-00020 (2003).

10 Pryor, K. O., Fahey, T. J., 3rd, Lien, C. A. & Goldstein, P. A. Surgical site infection and the routine use of perioperative hyperoxia in a general surgical population: a randomized controlled trial. *Jama* **291**, 79-87, doi:10.1001/jama.291.1.79 (2004).

11 Mayzler, O. *et al.* Does supplemental perioperative oxygen administration reduce the incidence of wound infection in elective colorectal surgery? *Minerva Anestesiol* **71**, 21-25 (2005).

12 Belda, F. J. *et al.* Supplemental perioperative oxygen and the risk of surgical wound infection: a randomized controlled trial. *Jama* **294**, 2035-2042, doi:10.1001/jama.294.16.2035 (2005).

13 Myles, P. S. *et al.* Avoidance of nitrous oxide for patients undergoing major surgery: a randomized controlled trial. *Anesthesiology* **107**, 221-231, doi:10.1097/01.anes.0000270723.30772.da (2007).

14 Meyhoff, C. S. *et al.* Effect of high perioperative oxygen fraction on surgical site infection and pulmonary complications after abdominal surgery: the PROXI randomized clinical trial. *Jama* **302**, 1543-1550, doi:10.1001/jama.2009.1452 (2009).

15 McKeen, D. M., Arellano, R. & O'Connell, C. Supplemental oxygen does not prevent postoperative nausea and vomiting after gynecological laparoscopy. *Can J Anaesth* **56**, 651-657, doi:10.1007/s12630-009-9136-4 (2009).

16 Bickel, A., Gurevits, M., Vamos, R., Ivry, S. & Eitan, A. Perioperative hyperoxygenation and wound site infection following surgery for acute appendicitis: a randomized, prospective, controlled trial. *Arch Surg* **146**, 464-470, doi:10.1001/archsurg.2011.65 (2011).

17 Thibon, P. *et al.* Effect of perioperative oxygen supplementation on 30-day surgical site infection rate in abdominal, gynecologic, and breast surgery: the ISO2 randomized controlled trial. *Anesthesiology* **117**, 504-511, doi:10.1097/ALN.0b013e3182632341 (2012).

18 Staehr, A. K., Meyhoff, C. S., Henneberg, S. W., Christensen, P. L. & Rasmussen, L. S. Influence of perioperative oxygen fraction on pulmonary function after abdominal surgery: a randomized controlled trial. *BMC Res Notes* **5**, 383, doi:10.1186/1756-0500-5-383 (2012).

19 Meyhoff, C. S., Jorgensen, L. N., Wetterslev, J., Christensen, K. B. & Rasmussen, L. S. Increased long-term mortality after a high perioperative inspiratory oxygen fraction during abdominal surgery: follow-up of a randomized clinical trial. *Anesth Analg* **115**, 849-854, doi:10.1213/ANE.0b013e3182652a51 (2012).

20 Stall, A. *et al.* Perioperative supplemental oxygen to reduce surgical site infection after open fixation of high-risk fractures: a randomized controlled pilot trial. *J Trauma Acute Care Surg* **75**, 657-663, doi:10.1097/TA.0b013e3182a1fe83 (2013).

21 Chen, Y. *et al.* Leukocyte DNA damage and wound infection after nitrous oxide administration: a randomized controlled trial. *Anesthesiology* **118**, 1322-1331, doi:10.1097/ALN.0b013e31829107b8 (2013).

22 Meyhoff, C. S., Jorgensen, L. N., Wetterslev, J., Siersma, V. D. & Rasmussen, L. S. Risk of new or recurrent cancer after a high perioperative inspiratory oxygen fraction during abdominal surgery. *Br J Anaesth* **113 Suppl 1**, i74-i81, doi:10.1093/bja/aeu110 (2014).

23 Kurz, A. *et al.* Effects of supplemental oxygen and dexamethasone on surgical site infection: a factorial randomized trial‡. *Br J Anaesth* **115**, 434-443, doi:10.1093/bja/aev062 (2015).

24 Wasnik, N. Role of supplemental oxygen in reducing surgical site infection in

acute appendicities: Our experience of sixty four cases. *International Journal of Biomedical and Advance Research* **6(02)**, 124-127 (2015).

25 Fonnes, S. *et al.* Perioperative hyperoxia - Long-term impact on cardiovascular complications after abdominal surgery, a post hoc analysis of the PROXI trial. *Int J Cardiol* **215**, 238-243, doi:10.1016/j.ijcard.2016.04.104 (2016).

26 Chiang, N., Rodda, O. A., Sleigh, J. & Vasudevan, T. Perioperative warming, oxygen, and Ilomedin on oxygenation and healing in infrainguinal bypass surgery. *J Surg Res* **220**, 197-205, doi:10.1016/j.jss.2017.06.043 (2017).

27 Kurz, A. *et al.* Supplemental oxygen and surgical-site infections: an alternating intervention controlled trial. *Br J Anaesth* **120**, 117-126, doi:10.1016/j.bja.2017.11.003 (2018).

28 Mayank, M., Mohsina, S., Sureshkumar, S., Kundra, P. & Kate, V. Effect of Perioperative High Oxygen Concentration on Postoperative SSI in Elective Colorectal Surgery-A Randomized Controlled Trial. *J Gastrointest Surg* **23**, 145-152, doi:10.1007/s11605-018-3996-2 (2019).

29 Kongebro, E. K., Jorgensen, L. N., Siersma, V. D. & Meyhoff, C. S. Association between perioperative hyperoxia and cerebrovascular complications after laparotomy-A post-hoc follow-up study. *Acta Anaesthesiol Scand* **63**, 164-170, doi:10.1111/aas.13235 (2019).

30 Alvandipour, M., Mokhtari-Esbuie, F., Baradari, A. G., Firouzian, A. & Rezaie, M. Effect of Hyperoxygenation During Surgery on Surgical Site Infection in Colorectal Surgery. *Ann Coloproctol* **35**, 9-14, doi:10.3393/ac.2018.01.16 (2019).

31 Ruetzler, K. *et al.* Supplemental Intraoperative Oxygen Does Not Promote Acute Kidney Injury or Cardiovascular Complications After Noncardiac Surgery: Subanalysis of an Alternating Intervention Trial. *Anesth Analg* **130**, 933-940, doi:10.1213/ane.0000000000004359 (2020).

32 Cohen, B. *et al.* Intra-operative high inspired oxygen fraction does not increase the risk of postoperative respiratory complications: Alternating intervention clinical trial. *Eur J Anaesthesiol* **36**, 320-326, doi:10.1097/eja.0000000000000980 (2019).

33 Ferrando, C. *et al.* Effects of oxygen on post-surgical infections during an individualised perioperative open-lung ventilatory strategy: a randomised controlled trial. *Br J Anaesth* **124**, 110-120, doi:10.1016/j.bja.2019.10.009 (2020).

34 Li, X. F. *et al.* Comparison of low and high inspiratory oxygen fraction added to lung-protective ventilation on postoperative pulmonary complications after abdominal surgery: A randomized controlled trial. *J Clin Anesth* **67**, 110009, doi:10.1016/j.jclinane.2020.110009 (2020).

35 Jiang, Q. *et al.* Supplemental Intraoperative Oxygen and Long-term Mortality: Subanalysis of a Multiple Crossover Cluster Trial. *Anesthesiology* **134**, 709-721, doi:10.1097/aln.0000000000003694 (2021).

36 Lin, X. *et al.* Intraoperative Oxygen Concentration and Postoperative Delirium After Laparoscopic Gastric and Colorectal Malignancies Surgery: A Randomized, Double-Blind, Controlled Trial. *Clin Interv Aging* **16**, 1085-1093, doi:10.2147/cia.S311190 (2021).

37 Park, M. *et al.* Perioperative high inspired oxygen fraction induces atelectasis in patients undergoing abdominal surgery: A randomized controlled trial. *J Clin Anesth* **72**, 110285, doi:10.1016/j.jclinane.2021.110285 (2021).

38 Reiterer, C. *et al.* Perioperative supplemental oxygen and NT-proBNP concentrations after major abdominal surgery - A prospective randomized clinical trial. *J Clin Anesth* **73**, 110379, doi:10.1016/j.jclinane.2021.110379 (2021).

39 Holse, C. *et al.* Hyperoxia and Antioxidants for Myocardial Injury in Noncardiac Surgery: A 2 × 2 Factorial, Blinded, Randomized Clinical Trial. *Anesthesiology* **136**, 408-419, doi:10.1097/aln.0000000000004117 (2022).

40 Akça, O. *et al.* Comparable postoperative pulmonary atelectasis in patients given 30% or 80% oxygen during and 2 hours after colon resection. *Anesthesiology* **91**, 991-998, doi:10.1097/00000542-199910000-00019 (1999).
